# Visual Search and Line Bisection in Hemianopia: Computational Modelling of Cortical Compensatory Mechanisms and Comparison with Hemineglect

**DOI:** 10.1371/journal.pone.0054919

**Published:** 2013-02-04

**Authors:** Linda J. Lanyon, Jason J. S. Barton

**Affiliations:** 1 Human Vision & Eye Movement Laboratory, Departments of Medicine (Neurology), Ophthalmology and Visual Sciences, Psychology, University of British Columbia, Vancouver, British Columbia, Canada; 2 International Neuroinformatics Coordinating Facility Secretariat, Karolinska Institutet, Stockholm, Sweden; Macquarie University, Australia

## Abstract

Hemianopia patients have lost vision from the contralateral hemifield, but make behavioural adjustments to compensate for this field loss. As a result, their visual performance and behaviour contrast with those of hemineglect patients who fail to attend to objects contralateral to their lesion. These conditions differ in their ocular fixations and perceptual judgments. During visual search, hemianopic patients make more fixations in contralesional space while hemineglect patients make fewer. During line bisection, hemianopic patients fixate the contralesional line segment more and make a small contralesional bisection error, while hemineglect patients make few contralesional fixations and a larger ipsilesional bisection error. Hence, there is an attentional failure for contralesional space in hemineglect but a compensatory adaptation to attend more to the blind side in hemianopia. A challenge for models of visual attentional processes is to show how compensation is achieved in hemianopia, and why such processes are hindered or inaccessible in hemineglect. We used a neurophysiology-derived computational model to examine possible cortical compensatory processes in simulated hemianopia from a V1 lesion and compared results with those obtained with the same processes under conditions of simulated hemineglect from a parietal lesion. A spatial compensatory bias to increase attention contralesionally replicated hemianopic scanning patterns during visual search but not during line bisection. To reproduce the latter required a second process, an extrastriate lateral connectivity facilitating form completion into the blind field: this allowed accurate placement of fixations on contralesional stimuli and reproduced fixation patterns and the contralesional bisection error of hemianopia. Neither of these two cortical compensatory processes was effective in ameliorating the ipsilesional bias in the hemineglect model. Our results replicate normal and pathological patterns of visual scanning, line bisection, and differences between hemianopia and hemineglect, and may explain why compensatory processes that counter the effects of hemianopia are ineffective in hemineglect.

## Introduction

Patients with homonymous hemianopia have lost vision in the field contralateral to their lesion, most frequently from damage to the optic radiations or striate cortex (V1), less commonly from damage to the optic tract or lateral geniculate nucleus. In contrast, patients with hemineglect have no visual loss but fail to be aware of or attend to objects and space on the side contralateral to their lesion. Hemineglect results typically from a lesion of the right posterior parietal cortex [1], [2], [3] but sometimes with frontal [4], [5], [6] or thalamic [7] lesions, and far less commonly from left-sided lesions.

Despite their visual loss, hemianopic patients direct more eye movements towards their contralesional blind side during visual search [2], [8]. In contrast, the scanpaths of hemineglect patients typically ignore contralesional space [2], [3]. During line bisection, when subjects have to indicate the middle of a line segment [9], hemianopic patients bias their perceived midpoint slightly towards the contralesional side [10], [11], [12], [13] whereas hemineglect patients make large bisection errors towards the ipsilateral side [14], [15]. Studies of eye movements during line bisection show that hemianopic patients cluster fixations at both the contralesional end of the line and a second central location just contralesional to the true midpoint, while hemineglect patients fail to explore the contralesional space and show a broad ipsilesional distribution of fixations [14], [15], [16]. Given the close link between overt attention and ocular fixations [17], these findings are consistent with a primary attentional failure for contralesional space in hemineglect patients, but a strategic adaptation to attend to the blind side in hemianopic patients. The processes by which hemianopic patients are able to adapt and preferentially attend to the blind side are still unclear.

Adaptation in hemianopia may require time. In fact, behaviour during the acute stage of hemianopia can resemble that of hemineglect, with subjects failing to explore and detect items in contralateral space [18]. However, adaptation can occur rapidly: in healthy subjects in whom hemianopia is simulated using gaze-contingent display, a strategic shift of fixations into the blind side develops over the first 5–7 trials [19]. Understanding the neural events behind the altered behaviours that adjust for hemianopia, and why they fail to do so for hemineglect, may help inform rehabilitation strategies.

We used a neurodynamic computational model of visual attention and eye movements [20], [21], [22] to examine the effects of candidate cortical compensatory processes in hemianopia after striate lesions and compare results to those obtained under conditions of simulated hemineglect following parietal lesions. This model is based on known response properties in retina and visual cortical areas. It has been used to accurately simulate attentional responses in individual cells in extrastriate, temporal and parietal cortices [21], and reproduce the scanning patterns of healthy humans and monkeys [22], as well as the pathological scan patterns seen after frontal and parietal lesions [20]. Since this model is biologically constrained, being based on known neurophysiological responses and connectivity, it provides a useful platform to examine the neural processes behind behaviour patterns in normal and pathological behaviour. We build on earlier work showing that a simulated parietal lesion in this model leads to scanning behaviours during visual search that are similar to those observed in hemineglect [20]. We now extend the model to examine and compare eye movements during visual search and line bisection tasks under conditions of damage to a striate cortex module, which we compare to a lesion to a parietal cortex module. While ocular motor behaviour likely incorporates top-down and subcortical (e.g. superior colliculus) contributions, our goal was to determine how much of the altered behaviour in hemianopia and hemineglect could be accounted for parsimoniously by known properties and hypothesized compensatory changes in activity in visual cortical areas, without need to incorporate additional factors. The results suggest possible mechanisms of cortical compensation that are available in hemianopia but prove ineffective in hemineglect.

## Methods

**Figure 1:**
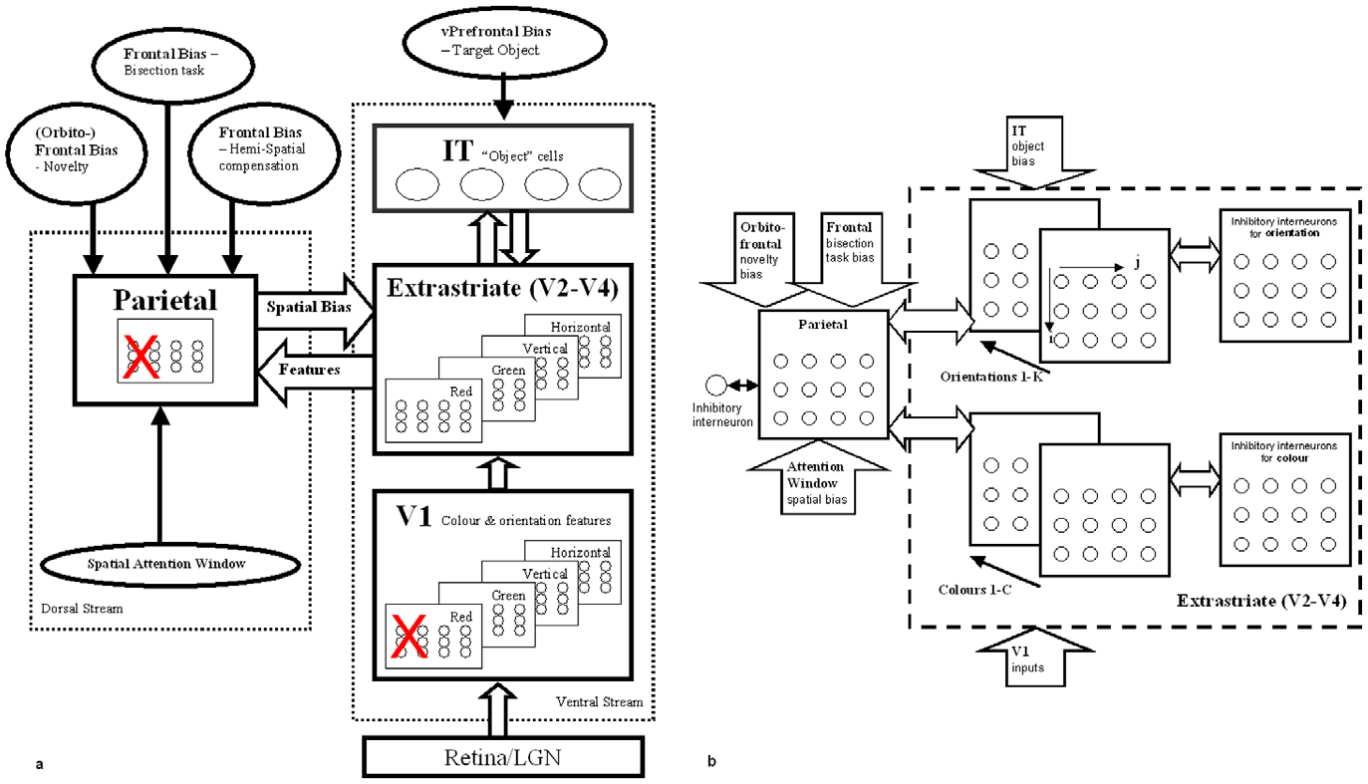
Model Architecture. a. Overview of the modules and connections. External biases to the system are shown as ovals. (Note that the actual number of cells in V1, extrastriate and parietal modules are more than shown. Typically several hundreds of neurons exist in each layer.) b. The inhibition and biases influencing competition in the extrastriate and parietal modules. These modules are reciprocally connected. Competition within the extrastriate module operates within each feature type at each spatial location. Neurons in the parietal module compete to determine which spatial location wins the competition for attention. Further description is given in the appendix.

Our computational model is an extension of the model of visual attention described by Lanyon and Denham [20], [21], [22] and is depicted in figure 1. The fundamentals of our non-lesioned model [21], [22] owe much to Gustavo Deco’s modelling of the dynamics of visual attention [23] and our V1 processing draws from the work of Steven Grossberg and colleagues [24]. According to a widely accepted neurobiological model that suggests cortical visual processing is performed in two main streams [25], [26], our model consists of a ventral and a dorsal stream, with interaction between the streams [27]. Each module within the extrastriate, temporal and parietal cortices consists of many neurons whose activity is updated at each time step, according to inputs from other neurons in the same module or in other modules, as well as bias signals that originate from brain regions external to the system (such as task-related frontal biases).

Processing in the retina consists of retinal ganglion broad-frequency-band cells performing simple centre-surround processing and retinal concentric single opponent cells processing colour information (see [21], [22]). Features are passed in a feed-forward hierarchical fashion from the retina to area V1 and then to ventral regions of the extrastriate cortex, which is modelled on responses and receptive field sizes in area V4 but which could encompass processing in area V2 also. The V1 and extrastriate areas encode features retinotopically with receptive field sizes being biologically realistic and larger in extrastriate cortex than V1 (see [22] for more details). These areas contain a retinotopic feature map for each feature represented (red & green colours and vertical & horizontal orientations). The extrastriate area feeds forward to area IT, which encodes invariant object representations. Within the ventral stream, biased competition operates between different features and different objects. During visual search, object-based attention results from a bias from frontal cortex (possibly ventral prefrontal area 46), which represents the search target, fed back to influence the competition between objects in IT. IT feeds back to the extrastriate module so that, as the target object wins the competition within IT, the feedback bias results in target features being enhanced and non-target features being suppressed in parallel across the extrastriate module.

In the dorsal stream, the model's parietal module is retinotopically organised and competition operates between different retinotopic spatial locations such that the location that becomes most active wins the competition to attract attention and, hence, becomes the new focus of attention. This is a simplified version of eye movement control processes conducted in the circuitry connecting the parietal eye field (monkey lateral intraparietal cortex), frontal eye fields and superior colliculus, The chosen fixation location is taken to be the centre of the winning parietal cell’s receptive field and a spatial transformation from the retinotopic parietal representation to a scene-based frame of reference is applied. It is assumed that such a transformation is performed either within parietal cortex or elsewhere in the saccadic decision processing stream. A ‘scan path’ of eye movements across the image is generated based on these series of fixations. Saccades are autonomously generated within the model when competition between objects within the ventral stream is resolved (using this model, Lanyon & Denham [21] produced simulated saccade onset times similar to those found in monkeys during visual search). The parietal module receives inputs from the ventral extrastriate module so that, as extrastriate cortex begins to represent the location of possible target features most strongly, these locations receive a favourable bias within the parietal module. Hence, possible target locations become enhanced and locations not containing target features become suppressed. The parietal module, therefore, acts as an indicator of behavioural relevance, as has been shown for the lateral intraparietal area in monkeys [28].

The parietal module receives an external bias that reflects the novelty of locations in the scene. This ‘novelty map’ is a scene-based representation indicating whether locations in the scene have been previously inspected and mediates inhibition of return [29], [30], [31] in the scanpath by contributing an excitatory bias to the competition in the parietal module. This could form part of a high-level search strategy that is controlled by frontal cortex. Under normal conditions the novelty bias to the competition in the parietal module causes the scanpath to be effective in exploring objects in the scene and not focusing in one hemifield or perseverating in any particular area [22]. However, when either the frontal novelty map or parietal cortex is damaged, symptoms of hemineglect and perseverance appear in the scanpath [20].

The model can operate in overt or covert attention modes. Here we report the overt mode in which eye movements are made and the model’s retina moves around the scene so that only the portion of the scene currently falling within the retina is available to the cortical system. This is more biologically realistic than a covert attention model when replicating eye movement behaviour, especially in the case of hemianopia where the blind field moves around the scene with fixation. The appendix contains a detailed description of the model. We restrict mathematical description in the main text to descriptions of the amendments to the V1, extrastriate and parietal modules for the lesions and our proposed methods of neural compensation.

### 1. V1 (Striate) Lesion

For hemianopic simulations we unilaterally lesioned the V1 module. To simulate a complete hemianopia we set the activity of all V1 neurons in one hemisphere to zero. In all simulations shown here the right hemisphere is damaged, resulting in left hemianopia.
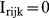
(1)where:

i ranges from 1 to m, m being the number of rows of neurons in V1.

j ranges from 1 to n/2, n being the number of columns of neurons in V1 i.e. an index of the hemispheric position of the neuron.

r ranges from 1 to 2 (i.e. all spatial resolutions are affected).

k ranges from 1 to K+C (i.e. affecting all orientations and colours).

where K is the total number of orientations and C is the total number of colours represented.

### 2. Parietal Lesion

We applied a unilateral right hemisphere lesion to the parietal module. In one set of lesion simulations the activity was set to zero across the complete hemifield, creating a step-function of impairment, analogous to hemispatial hypokinesia models of neglect [4], [32], [33], [34], [35]. In a second set of simulations, it was reduced by an amount that increased in a gradient fashion from the centre of the visual field towards the periphery, following some proposals that neglect is characterized by an anomalous attentional gradient [36], [37], [38]. We have used these models of parietal lesion previously to simulate hemineglect successfully [20].

For a complete hemifield (step-function) lesion:
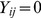
(2)


For a gradient lesion:
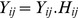
(3)


i ranges from 1 to m, m being the number of rows of neurons in the parietal module.

j ranges from 1 to n/2, n being the number of columns of neurons in the parietal module, again an index of the hemispheric position of the neuron.

H is the gradient of impairment matrix in which the values in each row increase linearly left to right from starting value 0.6, the maximum impairment, to 1, no impairment.

Elsewhere we have examined differences in scanpath behaviour during visual search of complex scenes under these two models of a parietal lesion [20]. For the line bisection task here, we will focus on results found with the step-function lesion, for simplicity, and then later compare to those produced using the gradient of impairment. We now describe the additions to the model that aim to provide a basis for compensation strategies used by patients with hemianopia following V1 lesion.

### 3. Spatial Compensation Bias in Hemianopia

With time and sometimes training, hemianopic patients make adjustments for their blind field. One compensatory strategy that has been suggested is a simple spatial bias in attention towards the blind field [14]. This concept also underlies rehabilitation strategies that use visuospatial cues to direct attention to locations in or at the edge of the blind field (e.g. [39]). We assume that an endogenous spatial attention bias develops gradually as a result of repetitive, spatially biased eye movement training. This bias is likely implemented over frontal-parietal networks that are associated with spatial attention. Here we model this bias as a spatial boosting signal applied to the parietal cortex that gives priority to locations within the blind hemifield.

The spatial compensatory bias is a step-function such that. 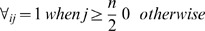
(4)where n = the number of columns of neurons in the parietal module Y

Hence, the value of the biasing input to a parietal neuron relates to the spatial position (i,j) of the neuron’s receptive field (a retinotopic parietal representation such as that found in monkey lateral intraparietal cortex, or a inter-parietal coordinate transformation, is assumed).

This bias is applied to the activity in the parietal module so that it evolves according to the following equation in place of the parietal equation in the appendix:
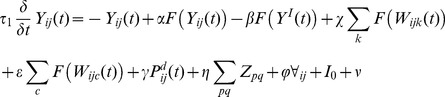
(5)where:


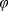
is the weight of the spatial compensation bias, set to 4.4 for visual search scenes, 0.9 for single line stimuli.

### 4. Perceptual Completion of Objects via Extrastriate Lateral Connections

In the study of eye movements by Barton et al. [14] hemianopic patients were able to target the “blind” portion of the horizontal line with their saccades. A compensatory mechanism such as the one described above may generate saccades into the blind hemifield, but how do the patients make accurate fixations to the unseen line rather than fixations randomly distributed in the blind hemifield? The blind portion of the line is not represented in V1 and hence activity related to the stimulus is not conveyed to extrastriate cortex. Therefore, the parts of the visual stream that are reliant on input from V1 are unaware of the retinotopic position of the line in the blind hemifield. Despite this, the eye movements of hemianopic patients suggest that the saccadic spatial decision-making process possesses fairly accurate position information about the likely location of the unseen parts of the line.

One possibility is that cells communicate to implement Gestalt-like grouping processes so that cells whose receptive fields are within the intact hemifield “prime” cells with receptive fields within the blind hemifield, implementing the expectation that the stimulus continues into the blind side. From monkey single cell recordings in V1 [40] we know that cells encoding segments of a line being traced by eye movements are preferentially excited compared to an unattended line, suggesting that cells that are sensitive to similar orientations communicate to enhance responses to collinearly oriented line segments. Further, in vivo optical imaging in other species has revealed anisotropic visual cortical connections that preferentially link neurons activated by contours that have similar orientation and are aligned collinearly [41], [42], [43]. Previous computational models [24] have simulated this type of lateral connectivity such that circuits in areas V1 and V2 enabled perceptual groupings over positions that do not receive visual inputs. This created collinear integration of line segments and illusory boundaries, such as the illusory perceptual contours in Kanizsa triangles. Similarly we model lateral connections in our extrastriate module in order to encode the expectation of continuation of the line into the hemianopic blind field. We assume that these connections are created from early learning of form and could conceivably be strengthened following rehabilitative training and visual experience after a lesion.

We model lateral connections in the extrastriate module such that neurons that encode orientation are connected with neighbouring neurons that have the same response properties. Neurons that have the same orientation tuning and whose receptive fields are retinotopically positioned to respond to a stimulus continuing in the direction of this orientation are preferentially connected to achieve collinear integration. The outputs of neighbouring neurons are convolved with an elliptical Gaussian kernel oriented along the axis of the direction of orientation preference. Note, this is similar to the long-range bipole-grouping kernels implemented elsewhere (layer 2/3 of V1 and V2 in [24]). Hence, surrounding neurons supply inputs to each other and the strength of these connections is such that inputs from retinotopically closer neurons are more highly weighted. Due to the nature of a hemifield loss either side of the vertical meridian, we model this process for horizontally tuned neurons. In reality, neurons tuned to many slightly different orientations could be involved in gathering information from the intact field but, for simplicity, we demonstrate this for horizontally selective cells only. In contrast to the elliptical kernels used elsewhere (e.g. [24]), we use only half of the horizontal kernel. The reason for this is that we are modelling a strengthening of the lateral connections in the direction towards the normal hemifield under conditions of hemianopia. Hence, we use a rightward-facing half kernel in order to simulate compensation for a left hemifield hemianopia. Our proposal is that these connections gather information from locations towards the intact field to strengthen responses of neurons located towards the blind field. In this way, collinear information is drawn from the good toward the poor visual fields. The elliptical Gaussian kernel is given by the following equation:
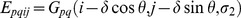
(6)where:

θ = 0 (horizontal orientation);

σ determines the width of the kernel along its long axis, set to 9;

δ determines the width of the kernel perpendicular to its long axis, set to 0.5.

**Figure 2:**
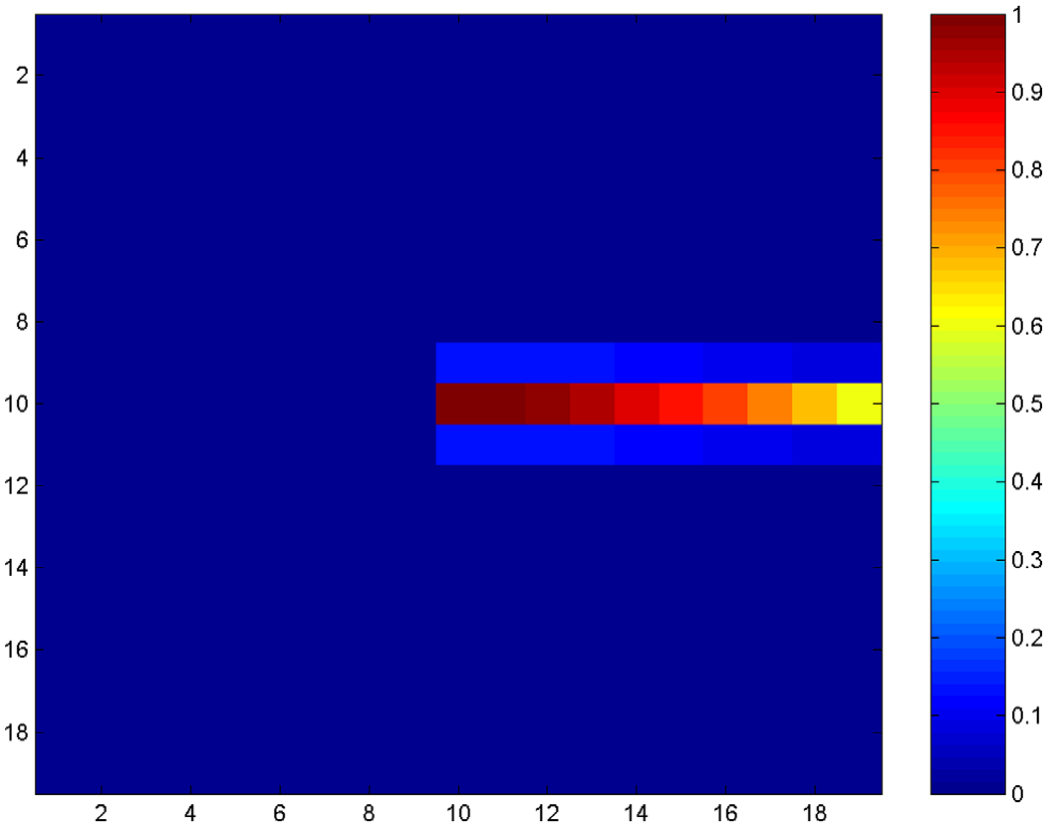
Kernel used to represent lateral connectivity in the extrastriate module. This kernel is convolved with the outputs of cells in the extrastriate cortex to give lateral inputs to horizontally selective cells. Hence, this kernel gives the weights of lateral inputs from neighbouring cells. The plot shows that inputs are strongest locally and horizontally towards the right, which is toward the intact hemifield in our simulations.

These parameters result in a rightward facing Gaussian of size 4×10, shown in figure 2.

The results of convolving neighbouring neuronal activities with this kernel are applied to the update of a horizontally selective neuron’s activity in the extrastriate module. The dynamics of neurons therein evolve as follows in place of the extrastriate equation for orientations in the appendix:

For k = 1 only (horizontal orientation):
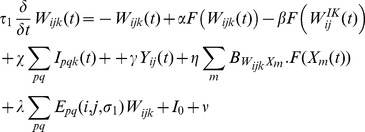
(7)where


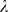
 is the weight of lateral inputs, set to 3.

### 5. Line Bisection Task

Visual search tasks in our model are driven by a top-down prefrontal bias related to the memory of the target object [20], [22]. In order to accurately simulate eye movements during line bisection, we introduced a top-down task-related bias that relates to the anticipated bisection point. At each fixation, information about the extent of the line is gathered and stored. This leads to an on-going estimate of the mid-point of the line that is used to bias eye movements, as detailed in the following sub-sections. After scanning, the line is bisected on the basis of this accumulated evidence and estimations of line extent. The line bisection procedure will be described after the process of gathering line end point estimations.

#### 5.1 Line End Information – V1 Endstopping

Hypercomplex cells in V1, known as endstopped cells, respond to the termination of a line [44]. In order to determine the extent of the line in our simulated line bisection task we extended our model to include V1 endstopping properties. The output from our V1 complex cell processes for horizontal orientation detection (I_ijk_ in appendix, where k = 1 for horizontal orientation) produces areas of high activity at the ends of a horizontal line (similar to a “brightness button”: [45]). We used this line termination information to provide a simplified form of V1 endstopping:

In V1 two matrices of endstopping cells exist: R and L, to represent the positions of the right and left line ends respectively.

A right endstop is determined when the activity to the left of the area of maximal I_ij1_activity is greater than that to its right.
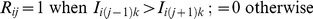
(8)where i,j = location of max(I_ijk_); k = 1;

A left endstop is determined when the activity to the right of the area of maximal I_ij1_activity is greater than that to its left.
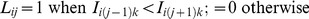
(9)where i,j = location of max(I_ijk_); k = 1;

This provides a direction-of-contrast sensitive line-end detection process. We assume that this information in V1 is used to inform higher-level cortical processes and, importantly, to gate extrastriate propagation of activity for an illusory line into a blind field. For example, when an endstopped cell sensitive to a left line-end is active within the current retinal view, this prevents extrastriate lateral connections propagating activity for the line to the left. Since this endstopping process originates in V1, it is affected by our simulated V1 lesion for hemianopia. This creates two possible viewing situations for fixations in hemianopia. First, if the line end is visible in the intact ipsilesional field then the line activity in extrastriate cortex is not propagated towards the blind side and a true representation of the left line end is perceived. Second, if the line extends into the blind hemifield where V1 endstopping cells are not available, then extrastriate activity propagates the perception of the line into the blind field and the position of the line’s end is not certain.

The V1 endstopped cells provide definitive information about the right and left ends of the line during scanning in a line bisection task. However, this information is not always available within the current retinal view and we suggest that other perceptual information about line length, based on the extent of cortical activity in higher cortical areas, is used to supplement this information. In fact, in order to simulate the bisection errors in hemianopia and hemineglect it was necessary to assume that the extent of cortical activity in the parietal cortex supplements the V1 endstopping information about the positions of the ends of line. If V1 endstopped cells alone are used then line bisection is more accurate in the simulated lesion states than is found in real patients. Why would patients use two sources of information rather than rely upon V1 endstopping information (or its higher cortical manifestation) alone? In hemianopia, V1 endstopping information from the contralesional line end is only present at eccentric contralesional fixations, beyond the end of the line. Hence, this information is not available during many other fixations made during scanning. In hemineglect, contralesional spatial representation and memory seem to be distorted. In this condition, the spatial memory of the locations of V1 endstopping that were discovered at some fixations during the scanpath might be impaired. Indeed, studies show that hemineglect patients sometimes make large ipsilesional bisection errors despite having scanned most of the line with their fixations [14]. Hence, V1 endstopping may not provide conclusive information under hemianopia and hemineglect, and supplementary perceptual information (from higher cortex) likely supplements the calculation of at least the contralesional line-end point.

Hence, we store the current estimate of the left and right line end at each fixation, as follows:

- If a V1 endstopped cell (left or right) is active within the current retinal view then this is stored.
- Otherwise, the furthest horizontal extent of cortical activation is stored. This is determined from thresholded levels of activity in the parietal module.

#### 5.2 line bisection

These line end estimates are stored over the course of several fixations and, at the end of the scanning, the mean right and left line end position is calculated from this series of estimates. The line is bisected at this mean position. This calculation is also performed on an on-going basis during scanning and the current estimate of the mid-point of the line is used to form a frontal cortex bias that is applied to the parietal cortex to influence saccadic attention capture, as detailed in the next section.

#### 5.3 bisection task bias

The location currently estimated to be the line midpoint is weighted highly in the competition for the capture of attention (and, hence, ocular fixations). This is achieved by applying an excitatory bias to the parietal module. The mnemonic bisection estimate is likely to be stored in scene- or object-based spatial memory. It is assumed that the necessary coordinate transformations take place within parietal cortex to convert this location into retinotopic coordinates for application to the parietal module here (our module represents a retinotopic area similar to monkey lateral intraparietal cortex). The form of the bias is a simple circular Gaussian:
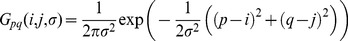
(10)


The Gaussian width parameter is set to: σ is set to 12. Location (i,j) is the current estimate of line centre.

This bias is applied to the activity in the parietal module so that it evolves according to the following equation in place of the parietal equation in the appendix:
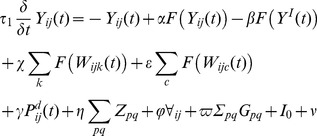
(11)where:


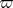
 is the weight of the bisection bias, set in the range 3 to 5 (Note that, generally when this bias is applied for a line bisection task, the spatial compensation bias, relating to search scenes, is not applied i.e. 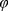 = 0 when 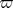
>0.).

### 6. Visual Stimuli

We use two types of stimulus display to replicate eye movement behaviours. First, for general visual search demonstrations, we use stimuli similar to those used in many feature-conjunction search tasks, in particular those by [46]: the scene consists of many vertically and horizontally oriented red and green bars in which the target is define by a unique conjunction of orientation and colour. We have previously shown that scanpaths produced by the normal intact model with these display conditions are similar to those produced by healthy humans [47], [48] and monkeys [46]. Second, for line bisection tasks, we simulate a single horizontal line (of size 45°×1°; 495×11 pixels) similar to that used for line bisection tasks in patients by Barton et al. [14]. Our model is able to accurately simulate stimulus size because the sizes of feature detection filters in V1 are matched with V1 receptive field size (see [22]). For consistency, initial fixations are placed at the centre of the display. Using these stimuli, we ran simulations of scanpath behaviour during visual search and line bisection, and the perceptual estimates of line centre for the latter under conditions of V1 or parietal lesion and no lesion.

## Results

As shown previously for normal visual search [22] the intact model explores the whole scene examining task-relevant stimuli, in a manner similar to healthy monkeys and humans. We now demonstrate that the intact model is also able to scan and accurately bisect a line like healthy humans during a horizontal line bisection task. Following a simulated V1 or parietal lesion, the model exhibits markedly different visual search and line bisection behaviours, which closely reproduce those seen in patients with real V1 or parietal lesions.

### 1. Acute Phase of Hemianopia Resembles Hemineglect

**Figure 3:**
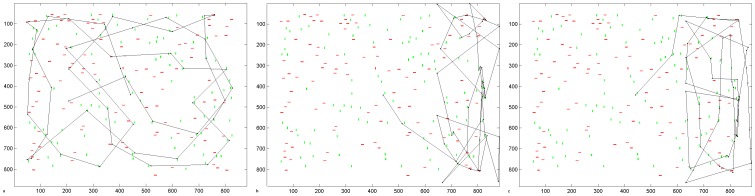
Visual Search Scanpaths Produced by the Model. Simulated scanpaths under (a) normal conditions, (b) hemianopic V1 lesion, and (c) hemineglect parietal lesion. No compensatory mechanism has yet been learned; the weight of the compensation bias is zero and the lateral connections in the extrastriate module are not yet present. Axes refer to pixel position in the scene.

Figure 3a shows a typical scanpath produced by the intact model in visual search. The scanpath targeted stimuli in the scene rather than blank areas and covered all quadrants of the scene. When the model sustained a V1 lesion resulting in a left hemianopia with no compensatory processes in place, the scanpath was altered (figure 3b): it favoured the intact ipsilesional (right) hemispace and omitted the blind side, similar to hemineglect scanpaths produced by the model with a parietal lesion (figure 3c). Hence, without adaptation, acute hemianopia resembles hemineglect in our model. While this may seem surprising, neglect-like visual behaviour has been found in patients at the acute stage of hemianopia after strokes [18].

**Figure 4:**
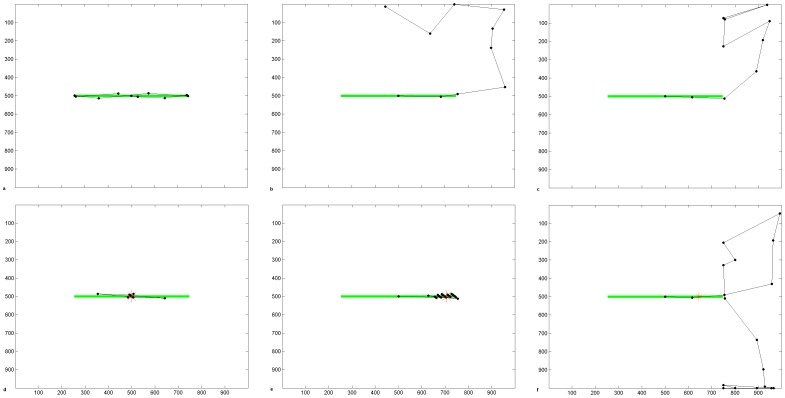
Line Bisection Scanpaths Produced by the Model. (a–c) Simulated scanpaths during free viewing of a horizontal line stimulus under (a) normal conditions, (b) hemianopic V1 lesion, and (c) hemineglect parietal lesion. (d–f) Simulated scanpaths during a line bisection task under (d) normal conditions, giving an accurate bisection point of 500, which is line centre, (e) hemianopic V1 lesion, giving a bisection point of 705 (18.6° offset, which is an ipsilesional error of 41.4% of line length), (f) hemineglect parietal lesion, giving a bisection point of 645 (13.2° offset, which is an ipsilesional error of 29.3%). The lines are depicted green, black dots are fixations, and dashed lines connect these to indicate the scanpath. Bisection points are shown as red asterisks. Axes refer to pixel position in the scene. The weight of the bisection task bias is 4.5 in these simulations.

Under free viewing conditions, to demonstrate the effects of stimulus-related capture of attention without a line bisection task, a single horizontal line was thoroughly inspected with the intact version of our model (figure 4a). Following a hemianopic V1 lesion the ipsilesional (right) of the line was attended first (figure 4b). While the left hemifield was often attended later in the scanpath, the left side of the horizontal line was never fixated. Following a parietal lesion, the model also neglected the left half of the line (figure 4c), and never entered left hemispace. The hemineglect scanpath had a tendency to move to the far right of the scene and to be unable to reorient to the line. This is partly a computational artefact caused by the unnatural scene boundary so that the intact parietal hemifield receives no input with which to reorient (as discussed in [20]), but is also partly symptomatic of the hemineglect condition.

During a line bisection task, we propose that healthy subjects and patients have a mnemonic task-related bias that biases their attention towards the object to be bisected, which helps maintain fixation at task-relevant locations. We will discuss the effect of the bisection task bias (equations 10 & 11) fully later but we introduce simulation results here that demonstrate scanning during a line bisection task in healthy, acute and adapted pathology. When we implemented the bisection bias, scanpaths for the intact condition (figure 4d) became similar to those from healthy people performing this task [14]: fixations clustered around the centre of the line and the line ends were only briefly explored. The line was bisected accurately at its centre, confirmed by 10 independent scanpath simulations. With both V1 and parietal lesions, scanpaths still ignored the contralesional line segment and there was an ipsilesional bisection error (figure 4e,f). Thus, the scanpath with the V1 lesion was still similar to that observed during acute hemianopia [18] and did not simulate the contralesional scanning and bisection error seen in chronic hemianopia [10], [11], [12], [14], [15], [16]. The average bisection point from 10 independent simulations in acute hemianopia was offset 17.4° ipsilesionally from centre (38.6% of line length). The average bisection point from 10 independent simulations of scanpaths in acute hemineglect was offset 12.1° ipsilesionally from centre (26.9% of line length). Note also that the top-down bisection task bias was insufficient to keep the scanpath on the line in hemineglect: we will return to this issue later. Thus the effect of the bisection bias is to focus fixations at the task-relevant location, but it does not generate any compensation for the initial ipsilesional bias in scanning and bisection judgments seen with an acute V1 or parietal lesion.

### 2. Spatial Compensation in Hemianopia versus Hemineglect

**Figure 5:**
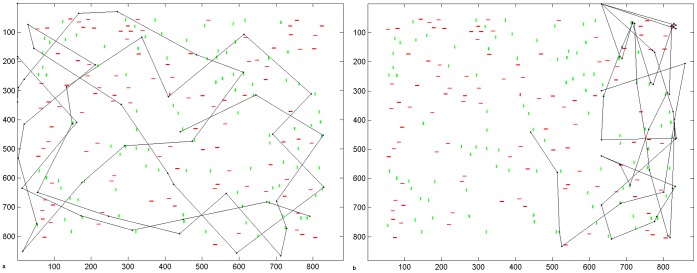
Effects of Spatial Compensatory Bias on Pathological Search Scanpaths. With the bias applied, the hemianopic scanpath is now able to search the blind hemifield (a). However, this bias fails to increase contralesional scanning in hemineglect (b). Axes refer to pixel position in the scene.

We propose that hemianopic patients learn to compensate for their blind field by biasing spatial attention towards it, as is suggested by studies of healthy subjects with simulated hemianopia [19]. To model this we applied a simple spatial compensatory bias to the parietal module. For visual search, figure 5a shows an example of a scanpath under hemianopic conditions (seen in figure 3b, but now with this spatial bias applied). The scanpath entered the left hemispace and most of the scene was examined. However, the hemianopic scanpath was slightly less effective compared to that with the intact model, in that there were more fixations in blank areas of the display: 54% of fixations were in blank areas and 46% near stimuli for the spatially compensated hemianopic simulation, compared to 5% in blank areas and 95% near stimuli for the control condition in figure 3a (average percentages from 10 independent scanpath simulations). If we applied this same spatial bias to the model with parietal lesion, it persisted in neglecting the left hemifield (figure 5b), similar to patient scanpaths [2]. Hence, this particular spatial compensation is ineffective under conditions of parietal hemineglect.

**Figure 6:**
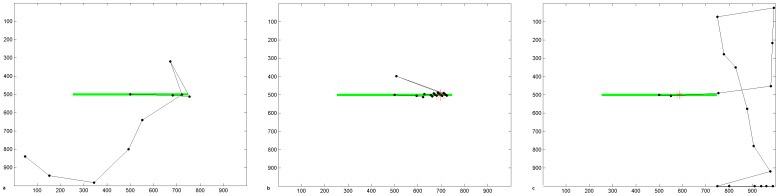
Line Scanning and Bisection with Spatial Compensation. (a–b) Hemianopic scanpath under (a) free viewing, where the model can scan the blind hemifield but does not accurately fixate the line and (b) with the line bisection task, where the task bias does bring the scanpath back to the target line, but only the ipsilesional line segment is viewed, and the bisection point is offset to this side (in this example position 697, a 17.9° offset, or ipsilesional error of 39.8% of line length). Hence spatial compensation alone does not reproduce real hemianopic performance for this task. (c) Hemineglect scanpath during a line bisection task. During this task and under free viewing (not shown), the contralesional line segment is severely neglected and the scanpath deviates from the line. The bisection point is shifted to the ipsilesional part of the line (in this example position 587, 7.9° offset, or an ipsilesional error of 17.6% of line length). This spatial bias is, again, ineffective in promoting contralesional scanning in hemineglect. Axes refer to pixel position in the scene.

While this spatial compensatory bias improved hemianopic scanpaths during visual search, it was less effective during line bisection. During free scanning, the spatial compensatory bias did increase contralesional fixations but could not place these on the contralesional line (figure 6a). This is due to the fact that there are no blind hemifield stimulus inputs from V1 to higher extrastriate and parietal areas to inform saccade planning. The addition of the bisection task bias (figure 6b) caused the scanpath to be more likely to remain on the ipsilesional line, due to the effect of trying to bisect a “seen” line, but failed to generate the compensatory increase in fixations of the contralesional line segment seen in real hemianopia (e.g. [14], [15], [16]). This indicates a need for another form of neural compensation in hemianopia, which we suggest is based on form completion processes that will be demonstrated in the next section. Last, with the hemineglect parietal lesion, the contralesional side remained neglected during line bisection as during visual search, despite the addition of the spatial compensatory bias (figure 6c).

### 3. Extrastriate Lateral Connections for Gestalt Grouping of Objects into Blind Field

**Figure 7:**
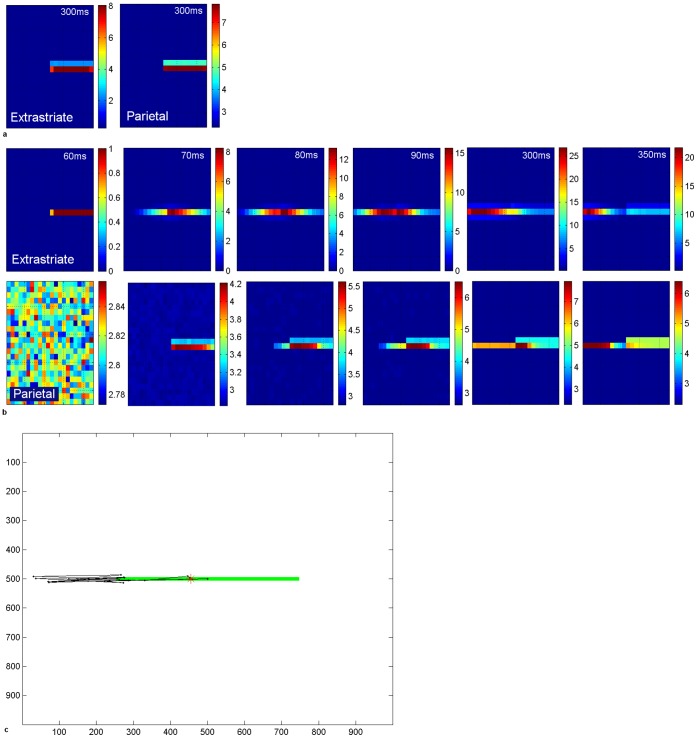
Cortical activity in hemianopia with and without extrastriate lateral connections. a. Acute hemianopia. Shows activity in the retinotopically-arranged horizontally selective extrastriate cells (left) and parietal (right) modules in the absence of lateral connections, at 300 ms post-fixation. Part of the line is represented in the normal hemifield but the blind hemifield is inactive due to lack of V1 inputs, despite the fact the line actually extends across the whole retina at this fixation point. Since only the intact hemifield contains stimulus-related activity in the parietal module, the next location to attract attention will be on the line in this hemifield. This reflects acute hemianopia before any rehabilitation training. Level of activity is given by the coloured bar. b. Hemianopia with perceptual completion. The top row shows activity in the extrastriate module for the cells selective for horizontal orientations, when lateral connections are enabled. Subplots show activity at 60 ms (onset of the stimulus-related response), 70 ms, 80 ms, 90 ms, 300 ms and 350 ms after fixation at the centre of the horizontal line. The bottom row shows the associated activity in the parietal module. Over time, the left of the line becomes most strongly represented and, therefore, will be chosen as the next saccade target. Note that levels of activity become high in extrastriate and parietal cortices for the blind hemifield. Level of activity is given by the coloured bar. c. Hemianopic scanpath with extrastriate lateral connections. The bisection point, which is offset contralesionally to the midpoint (position 455: −4.1° offset, which is a contralesional error of 9.1% of line length), is shown as a red asterisk. In this simulation the bisection task bias weight was 3 and no spatial compensation was involved. Axes refer to pixel position in the scene.

We now introduced extrastriate lateral connections that may compensate for the loss of bottom-up visual inputs, using Gestalt-like form completion. These have the effect of propagating cortical activity for the line into the blind hemifield (figure 7a,b). Hence, despite the lack of V1 input in the blind hemifield, a “virtual” line is represented in the extrastriate module. Activity propagates along the line from the normal to the blind hemifield in the extrastriate module during the period after the onset of the stimulus-related response. Notice that levels of activity in extrastriate cortex are higher when lateral excitation is in effect (compared to level of extrastriate activity in figure 7a at 300 ms post-fixation, levels in figure 7b at 300 ms are around 3 times higher). Ventral-to-dorsal stream connections convey this information from the extrastriate module to the parietal module and biases the competition for attention there. Hence, over time, as the contralesional segment of the line becomes most strongly represented, a location in that hemifield will be chosen as the next saccade target. This explains the tendency for hemianopic patients to make more saccades to the contralesional line segment and much fewer to the ipsilesional segment [14], [15], [16].

A typical simulated scanpath for hemianopia with this lateral connectivity is shown in figure 7c. As a result of the propagated cortical activity along the line into the blind side, the scanpath accurately targeted the blind portion of the line, even though it was not represented in V1 and hence higher cortical areas received no “bottom-up” stimulus information from V1. Figure 7c also shows that the scanpath tended to overshoot the blind end of the line before returning back to the line end and repeating this pattern a few times. This pattern also has been observed in hemianopia [14]. Such a scanpath probably allows the person to confirm the contralesional line end. In our model it is caused by extrastriate activity being propagated into the blind hemifield and not halted by V1 endstopping during fixations in which the line extends into the blind hemifield. When the end of the line is visible in the intact field (at far left fixation here), line termination information is available from V1 endstopped cells and extrastriate line propagation is halted. Hence, for some fixations line end information is available while for others it is not: in the latter case the line length perceptual estimate comes from the extent of activity in cortex. The pooling of these estimates leads to a small *contralesional* bisection error (figure 7c), as seen in real hemianopia [11].

**Figure 8:**
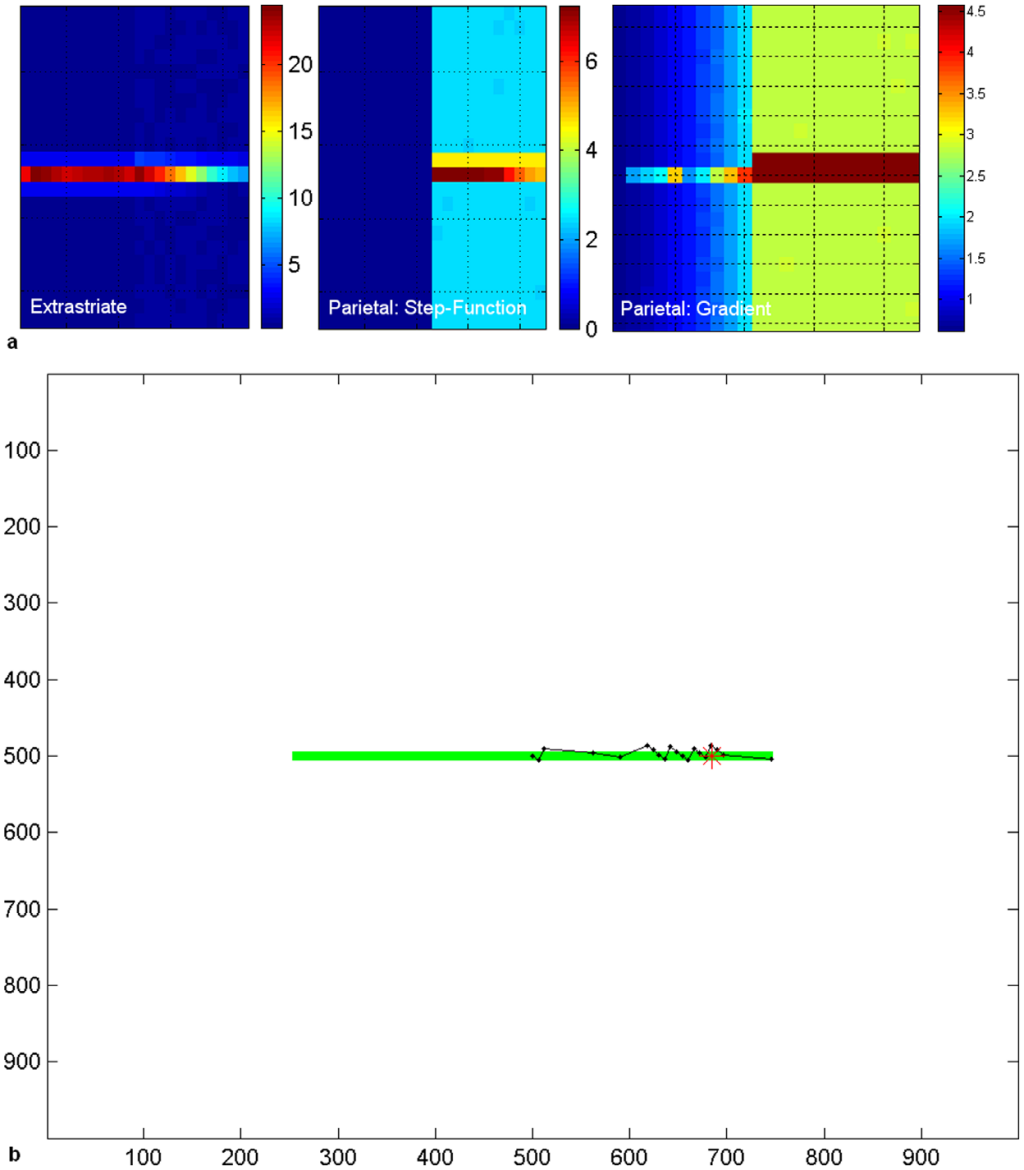
Failed Perceptual Completion in hemineglect with extrastriate lateral connections. a. Cortical activity at 300 ms post-fixation is shown in the extrastriate (left panel) and parietal (middle panel) modules when the parietal module has a step-function lesion. Activity for the entire line is present in the extrastriate cortex and the contralesional part of the line is reinforced by compensation through lateral connections. However, this increased extrastriate activity fails to activate a parietal representation of the contralesional line and, hence, this is neglected. If the parietal module is lesioned in a gradient fashion instead (right panel), the increased extrastriate activity does result in some activity in the damaged parietal area but this is insufficient to win the competition for attention. Level of activity is given by the coloured bar. b. Scanpath with parietal lesion (step-function lesion) and extrastriate lateral connections. The bisection point, which is offset into the ipsilesional half of the line (position 685∶16.8° offset, or an ipsilesional error of 37.4% of line length), is shown as a red asterisk. In this simulation the bisection bias weight was 5. Axes refer to pixel position in the scene.

With the parietal lesion simulating hemineglect, extrastriate lateral connections did not increase contralesional scanning or reverse the ipsilesional bisection error. Here, the extrastriate module receives V1 inputs from both hemifields and is thus activated along the entire line segment (figure 8a). The lateral connections increased contralesional line activity in extrastriate cortex, but this increase failed to activate a parietal representation sufficiently for the contralesional line segment to capture attention: hence the ipsilesional line segment was scanned but the contralesional segment was neglected (figure 8b). This resulted in a large ipsilesional bisection error. This is due to the fact that, even if contralesional V1 endstopping information was available during the first central fixation, subsequent fixations move the retina to the right and lead to a truncated representation of the left of the line in parietal cortex. In our model, this extent of the perceptual experience of the line in the parietal module conflicts with any early record of the actual line end (based on V1 endstopping) which subsequent fixations fail to confirm, due to lack of scanning on the left. The uncertainty in the left line-end decision-making process is biased by the greater amount of information gathered from fixations to the right which, based on the extent of cortical activity for the line, gave a perceptual experience of a longer line to the right. Even application of the spatial compensatory bias and the extrastriate lateral connections together had no effect in lessening the neglect of the left hemifield following a parietal lesion in our model.

### 4. Effect of Bisection Task Bias

**Figure 9:**
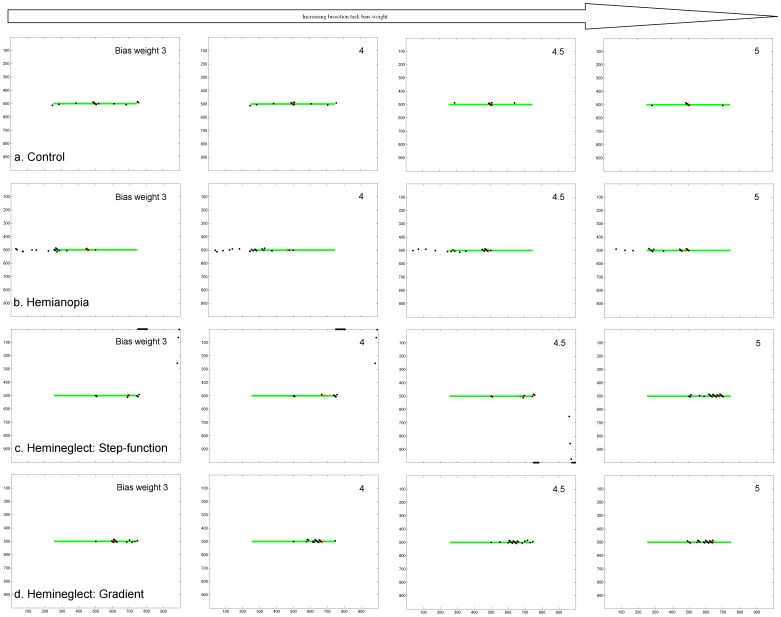
Example Scanpaths Showing the Effect of Lesion Condition and Bisection Task Bias on Line Scanning & Bisection. Each sub-plot shows the position of scanpath fixations (black dots) and point of line bisection (red asterisk) under various healthy and lesion conditions when the bisection bias weight was systematically raised, being set to the following values in order left to right: 3, 4, 4.5, 5. Axes refer to pixel position in the scene. a. Intact model. Raising the weight of the bisection task bias has the effect of concentrating the scanpath at the line centre. Bisection was consistently at line centre, position 500 (zero error). b. V1 lesion model. Raising the weight of the bisection task bias causes more fixations to be clustered around the location, offset contralesionally to line centre, which will later become the bisection point. As the task bias weight increases, less fixations are placed at the contralesional line end, but this is still where the majority of fixations are made. Bisection points were always contralesionally offset, in these simulations, from left to right, being: 455 (−4.1° offset, 9.1% of line length error), 468 (−2.9° offset, 6.5% error), 488 (−1.1° offset, 2.4% error), 482 (−1.6° offset, 3.6% error). c. Parietal lesion model using a step-function. If the weight of the bisection task bias is weak, the scanpath does not stay on the line. Bisection points were always highly ipsilesionally offset, in these simulations, from left to right, being: 688 (17.1° offset, 38.0% of line length error), 680 (16.4° offset, 36.4% error), 674 (15.8° offset, 35.2% error), 681 (16.5° offset, 36.6% error). d. Parietal lesion model using a gradient. Bisection points again were highly ipsilesionally offset, in these simulations, from left to right, being: 611 (10.1° offset, 22.4% of line length error), 669 (15.4° offset, 34.1% error), 652 (13.8° offset, 30.7% error), 644 (13.1° offset, 29.1% error).

**Figure 10:**
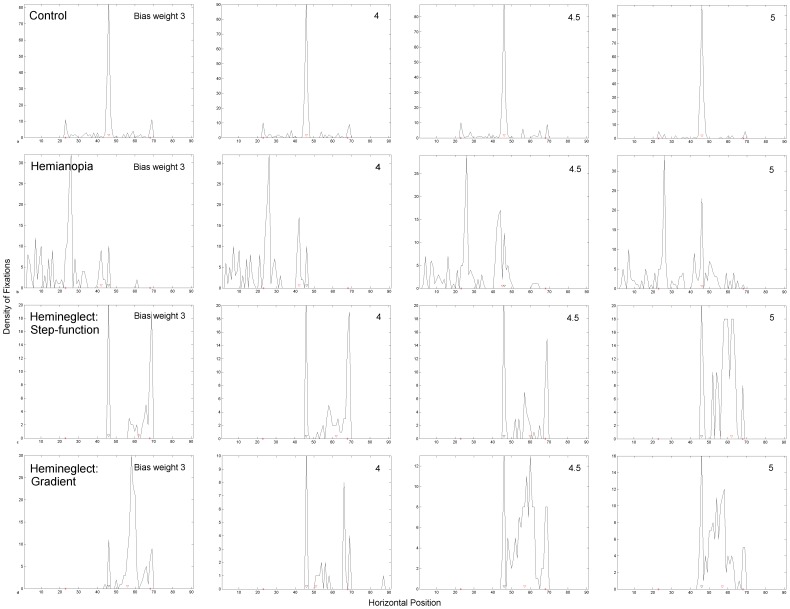
Fixation Density Plots from Multiple Scanpath Simulations. This shows the effect of lesion condition and bisection task bias on line scanning & bisection. Results come from 10 independent scanpath simulations for each condition. The density of fixations at each horizontal position across the scene is plotted in 1° bins. Note that, in order to clearly show each plot, sub-figures are plotted to the scale dictated by the Y axis maximum and this varies between plots. The control condition is shown in row (a), V1 lesion in row (b), parietal step-function lesion model in row (c) and parietal gradient lesion model in row (d). As in figure 9, the bisection bias was systematically increased here such that it is set to 3 in the leftmost column, 4 in the second column, 4.5 in the third and 5 in the final column. Fixations that landed more than 1° away from the line in the vertical direction are excluded. This only affected parietal lesion simulations where fixations were eliminated when they went off the line towards the edge of the scene. The number of simulations in which this occurred is given in table 1.

Figures 9 and 10 and table 1 show that the line bisection task bias has the effect of causing more fixations to be focused at task-relevant locations. Figure 9 shows examples of individual scanpath simulations and figure 10 shows the fixation density of fixation at each horizontal position along the line from 10 independent scanpath simulations, which can be compared to similar plots in patients and also gaze-contingent simulations of hemianopia [14], [49]. In the control simulations (figures 9a, 10a), raising the weight of this bias tends to focus fixations at line centre rather than scanning the entire line, just as healthy humans do [14]. When the bias is weighted less strongly, perhaps reflecting less concentration on the task, more fixations are placed along the periphery of the line, with no preference for left or right. The line is always bisected accurately in the healthy simulations.

**Table 1:** Comparison of bisection errors under different lesion conditions whilst varying the weight of the bisection bias.

Lesion Type	Weight of Bisection TaskBias	Bisection Point (rounded to nearest pixel position) 500 is actual line midpoint	Offset	Bisection error (as percentage of line length)	Percentage of neglect scan paths that stray off the line and explore blank regions in the right of the image.
Control	**3; 4; 4.5; 5**	500	0	0	Always on line
V1 Left Hemianopia					
	**3**	461	−3.6°	−7.9%	All hemianopia scan paths stay on the line and the leftward virtual continuation of the line.
	**4**	460	−3.7°	−8.2%	
	**4.5**	484	−1.4°	−3.2%	
	**5**	499	−0.06°	−0.1%	
Parietal: step-function					
	**3**	672	15.6°	34.7%	100%
	**4**	681	16.5°	36.6%	90%
	**4.5**	654	14.0°	31.1%	100%
	**5**	678	16.2°	36.0%	10%
Parietal: gradient					
	**3**	609	10.0°	22.1%	30%
	**4**	555	5.0°	11.1%	100%
	**4.5**	621	11.0°	24.5%	40%
	**5**	617	10.6°	23.6%	40%

10 independent scan paths were simulated for each condition.

Under hemianopic conditions (figures 9b, 10b) fixations cluster at two locations: the contralesional end of the line and around a central location, offset slightly contralesionally from the line midpoint, which will later become the bisection point. Hence, the ipsilesional segment of the line is inspected less. When the line bisection task bias is weak most fixations are located at the blind end of the line. Strengthening the line bisection task bias causes more fixations to be clustered around the central bisection point but with most fixations still being placed at the line end. This replicates very well the line bisection scanning behaviour observed in hemianopic patients [14] and in simulated gaze-contingent hemianopia in healthy controls [49].

Under hemineglect conditions (figures 9c,d, 10c,d), the contralesional hemifield is consistently neglected. If the weight of the line bisection task bias is weak, the scanpath strays from the line and cannot re-orient to it. This differs slightly from patient behaviour in which fixations tend to stay on the ipsilesional line (e.g. [14]). To simulate real hemineglect scanning for this task, the line bisection task bias must be weighted even more strongly than in hemianopic or normal simulations. This suggests that a brain with a parietal lesion requires a stronger top-down task-related bias to maintain task-relevant fixations, compared to hemianopic and control subjects.

### 5. Line Bisection Point

In our lesion simulations the extent of the line is determined by a combination of the V1 endstopping process and by the extent of the cortical activity invoked by the line and extrastriate lateral propagation. With this method, table 1 shows that our simulated bisection errors are similar to those found in patients viewing the same line length [14]. In our simulated left hemianopia bisection points were slightly contralesionally offset by –0.1 to –3.7°, which compares favourably with the contralesional error of –2.8 (+/−2.5) reported in hemianopic patients [14]. In our simulated left hemineglect, bisection points were markedly offset ipsilesionally, by 5.0 to 11.0° in the gradient model, and 14.0 to 16.5° in the step-function model. This also compares favourably with the ipsilesional error of 6.3° (+/−5.5) reported for patients with hemineglect [14], particularly for the gradient model.

If the V1 endstopping process alone was used to determine the bisection point, scanpaths were similar to those shown in figure 9 but the simulated bisection points were placed close to the centre of the line in our lesion conditions, replicating healthy rather than pathological perception of line length and bisection behaviour.

## Discussion

We used a neurophysiologically-based computational model to examine the role of possible cortical neural compensatory processes in hemianopia and compared results to those obtained under conditions of simulated hemineglect. We simulated two different tasks: visual search and line bisection, in the latter using an additional top-down task-related bias in which attention was biased by the spatial location of the ongoing estimate of line centre. Our modelling produced several findings. First, we showed that the model reproduced the scanning patterns and perceptual results of healthy human subjects. Second, when the V1 module was lesioned to simulate hemianopia, behaviour initially resembled hemineglect, but the addition of plausible compensatory attentional biases and also strengthening of extrastriate form completion processes replicated the fixation distributions seen in hemianopic visual search and line bisection. We found that the small contralesional bisection error seen in hemianopia was linked to the extrastriate form completion process, which influenced cortical perception of line length, and that the activity of V1 endstopping cells alone could not account for pathological traits in judging line length. Finally, we showed that, when the parietal module was lesioned to simulate hemineglect, the model reproduced the abnormal ipsilesional scanning bias observed in patients. Cortical representations of line length were affected by the parietal lesion and reproduced the large ipsilesional bisection errors typical of hemineglect. Scanning and bisection errors typical of hemineglect persisted even after the introduction of all compensatory processes. Thus, this model reproduces many of the clinical features seen in both chronic hemianopia and hemineglect, and suggests a neural basis for some compensatory processes that are operative in hemianopia but ineffective in hemineglect.

### 1. Acute Hemianopia

At the acute stage – i.e. without compensatory processes - behaviour of our V1-lesioned hemianopic model resembled that of hemineglect: scanpaths ignored the contralesional hemifield and there was an ipsilesional line bisection error. Similar visual behaviours have been observed in the acute stage of real hemianopia [18], [50]. However, both hemianopic patients and healthy subjects with virtual hemianopia adapt to their field loss and compensate with more fixations in the hemispace on their blind side, and in line bisection show a small contralesional bisection error. The key challenge for our model was to determine what neural mechanisms might underlie the adjustments seen in hemianopia. To explore this we introduced two neurophysiologicaly plausible compensatory strategies into our lesion models: a contralesional spatial attention bias, and an increase in mechanisms for form completion in extrastriate cortex.

### 2. Spatial Compensatory Bias in Hemianopia

Rehabilitative strategies that use visuospatial cues to enhance contralesional attention have been shown to ameliorate the effect of visual field defects [50], [51]. We modelled a simple spatial bias that reflected the neural mechanisms of biasing spatial attention towards the blind field. This spatial compensation was intended to represent an endogenous neural signal that could be linked to repetitive eye movements or spatial attention training. This bias could have several origins. One could be prefrontal cortex, reflecting a cognitive strategy to bias attention to the blind field. Since rehabilitative training typically involves encouraging eye movements toward the blind field or its periphery, one might speculate that adaptation of spatial attention is based in eye movement control circuitry, since processes of spatial attention and eye movement planning share at least some common circuitry [52], [53], [54], including the parietal, frontal and supplementary eye fields (particularly in the right hemisphere [55], [56]) as well as the superior colliculus. More speculatively, the spatial attention bias could reflect processes operating at lower levels of the visual hierarchy that attentionally boost “bottom up” visual signals from the blind hemifield to areas other than V1, such as parietal cortex: a candidate for this could be attentional gating at the level of the pulvinar [57]. Regardless, this compensatory bias increased scanning of contralesional hemispace during both visual search and line bisection. Similar to that in real hemianopia [50], [58], visual search in contralesional hemispace was inefficient: many fixations were placed in blank areas of the scene rather than on stimuli. The spatial compensatory bias alone did not reproduce line bisection scanning as successfully: fixations failed to land on the contralesional line segment, in distinction to what occurs in real and virtual hemianopia [14], [49].

### 3. A Role for Extrastriate Lateral Connectivity

To explain how hemianopic patients [14] and healthy controls with virtual hemianopia [49] accurately locate the contralesional segment of a line that extends into the blind hemifield, we considered a contribution from extrastriate form completion processes. We tested this in our model using lateral connectivity between neurons tuned to a similar orientation preference, which has been demonstrated neurophysiologically [41], [43] and implemented in previous computational models [24]. We suggest that these extrastriate lateral connections, which exist in the pre-lesion state, are strengthened as an adaptation to the loss of “bottom-up” visual inputs, such that neurons preferentially connect to other neurons whose receptive fields are positioned towards the normal hemifield. Hence, perceptual information is “filled in” from the intact towards the blind hemifield. Thus, when an object in the intact field extends beyond its border with the blind hemifield, perceptual activity propagates into the blind hemifield, creating a ‘virtual’ representation of the object continuing into the blind side. It is important to emphasise that our suggestion involves a strengthening of lateral connectivity as a result of adaptation to field loss. This provides more powerful contralesional form completion processes than those normally found in the healthy brain. Our hemianopic model extrapolates form into the blind field despite the absence of collinear information in the blind field, which would normally be required in the healthy brain to achieve continuation of form. Hence, this one-sided stimulus completion contrasts with results from studies of healthy people and monkeys and associated models that require collinear line information at both ends of a perceptual gap in order to complete an illusory boundary [24], [59].

These strengthened extrastriate lateral connections resulted in fixations accurately landing on unseen contralesional line segments during a line bisection task in hemianopia. Despite lack of V1 inputs for the segment of the line in the blind hemifield, over time this became very strongly represented in extrastriate and parietal cortices due to this propagation and our model’s ventral-to-dorsal stream connection. As a result, contralesional locations on the line tended to attract attention. This can account for the tendency of hemianopic patients to make more fixations on the contralesional portion of the line [14], [15], [16]. As in real [14] and simulated hemianopia [49], our simulations produced scanpaths where fixations clustered at two locations: most on the contralesional line-end, and several at a location slightly offset contralesionally from true line centre, which later became the bisection point.

As an aside, we note that this aspect of our model may also explain the phenomenon of “*hemianopic completion*” [60], whereby some patients with hemianopia report a retinotopic completion of form when the object overlaps the vertical boundary of their field defect [60], [61], [62]. Poppelreuter [63] observed that some patients with hemianopia reported seeing an entire figure when only part of the presented form fell in the intact visual hemifield. Half-circles and half-stars in the intact hemifield were also completed, showing that residual vision in the hemianopic field was not the cause. In the same way, propagation of activity in our model’s extrastriate cortex continues into the blind field when the left end of the line extends to the border of the blind hemifield. In Poppelreuter’s study, regularly shaped objects such as circles and squares were frequently completed but irregular figures were not. From our model, we predict this is due to a rather simplistic completion of form by collinear integration achieved by cells tuned to similar orientations, with neighbouring receptive fields exciting one another. Complex shapes in which the angle of the form changes frequently cannot elicit the necessary lateral excitation in extrastriate cortex. As another example, Kasten and Poggel [64] reported a hemianopic woman in whom objects presented and perceived in her intact hemifield were transposed into the blind field. Of note, the hallucinatory objects in the blind hemifield were not coloured, despite good colour vision in the intact hemifield. In our model, the lateral connectivity that propagates perception into the blind field operates in the domain of orientation selectivity and not the colour domain: that is, cells that are tuned to similar (same) orientations excite one another within a neighbourhood region, but cells tuned to particular colours do not. Hence, our model predicts that form completion in the blind hemifield would be achromatic. Hemianopic completion is not found in all hemianiopic patients and we attribute this to individual differences in neural adaptation: the weights of these lateral connections in some people become sufficiently high to overcome the requirement that boundary completion requires a post-gap collinear stimulus for an illusory boundary to form, while in others the strength of connections remain at nearer-normal level and are insufficient to produce full illusory form completion into the blind field.

It is not totally clear whether callosal connections mediate the collinear connections between cells representing locations close to the vertical meridian. Early studies in cats suggest that callosal connections may mediate transmission of information across the vertical meridian so that regions of areas 17 and 18 that represent locations near the vertical meridian are interconnected, whereas cells representing more peripheral parts of the visual field are not interconnected [65], [66]. Later studies suggested that long-range callosal connectivity exists and confirmed that this is orientation-specific, at least for cat areas 17 and 18 and macaque areas V1 and V2, leading to suggestions that callosal connectivity may achieve the same goals as intra-hemispheric long-range horizontal projections [67], [68], [69]. In contrast, Bosking et al. [70] found that callosal connectivity tends to be non-specific for orientation in the tree shrew. However, in this species, both visual fields were found to be represented in each hemisphere, with the ipsilateral visual field being highly compressed compared to the contralateral field. In this situation, the integration of visual information across hemifields could be achieved by unilateral connectivity within one hemisphere rather than requiring callosal interaction. The results of our modelling suggest the importance of orientation-specific callosal projections. This finding warrants further neurobiological investigation in primate species closest to humans to establish the extent and orientational specificity of callosal projections that innervate extrastriate cortex.

Possibly supporting this suggested extrastriate involvement in form completion in hemianopia, functional magnetic resonance imaging (fMRI) of hemianopic patients before and after saccade training shows that, immediately after training, increased activity is found in the extrastriate cortex of the lesioned hemisphere [39], [71]. In our simulations we see increased levels (by around 3 times) of extrastriate activity during propagation of activity into the blind field due to lateral excitation compared to the situation when these lateral connections are not in effect (figure 7). Recent neuroimaging has also revealed increased activity in extrastriate cortex during the hemianopic completion phenomenon [72]. Although this does not constitute proof of our mechanism, it is possible to speculate that one source of this enhanced extrastriate signal is increased extrastriate lateral connectivity propagating activity into the blind hemifield.

### 4. Hemineglect

In contrast to the adaptation in hemianopia, a strong spatial compensatory bias in the parietal lesion model did not improve hemineglect in line bisection or allow the scanpath to attend the whole scene during visual search, similar to patient scanpaths [2], [14]. Since our spatial attention bias was applied to the parietal cortex, which was lesioned in the hemineglect condition, it is not surprising that it was ineffective. However, of interest is the fact that the comparative simulations of hemineglect and hemianopia demonstrate that the attentional bias created as a result of spatial rehabilitative training must be applied to (or sourced from) a region, or network of regions, that is damaged in hemineglect and not in hemianopia. Our simulated parietal lesion likely corresponds to a human lesion of the right posterior parietal cortex [1], [2], [3] but could also be associated with frontal [4], [5], [6] or thalamic [7] regions linked with hemineglect. Our simulations suggest that these regions must be implicated in the spatial attention biasing that proves effective in hemianopia but ineffective in ameliorating symptoms of hemineglect. Frontal-parietal circuitry is known to be involved in attentional processing and damage to certain parts of this circuit cause hemineglect [73]. This circuit remains intact in hemianopia and, therefore, our spatial attention bias routed through these circuits is effective in that condition. This allows our hemianopic simulations to explore the contralesional visual field and hemianopic patients to benefit from spatial attention training. The difference in the location of damage with respect to the circuitry involved in spatial attentional biasing explains why rehabilitation strategies used for hemianopia, such as spatial cueing to the contralesional space, are less effective in hemineglect (see [74], [75] for reviews). Hemineglect can be briefly improved by an exogenous spatial cue to the neglected field but this benefit does not transfer endogenously to create sustained adaptation of attention to the neglected field [76]. A likely explanation is that the exogenous capture of attention involves “bottom-up” neural networks (thalamic-primary visual-extrastriate) that remain intact in hemineglect but endogenous attention requires a thalamic-frontal-parietal network that is damaged and thus cannot support the transfer of training to a sustained endogenous state. In contrast, long-term neural adaptation appears to be possible in hemianopia, allowing hemianopic patients to preferentially bias attention to their blind field because the regions associated with endogenous attention are intact in these patients. The lack of success of rehabilitative eye movement and attentional cuing in hemineglect is also due to the heterogeneity of the population: physiological abnormalities differ widely between patients and sub-types of neglect are not well established.

While the lateral connections in our extrastriate module generated strong extrastriate activity for the contralesional line in both hemineglect and hemianopic simulations, this did not bias processing in the lesioned parietal module enough to attract attention to the contralesional side, and the representation of the line that developed in the model’s parietal cortex was truncated contralesionally. This cortical under-representation of contralesional line length was exacerbated by the scanpath favouring the ipsilesional segment of the line. The cortical perception of line length biased the overall length judgement that is determined from this information combined with endstopping information from V1. Hence, even with these additional compensatory processes, our hemineglect line bisection simulations still resulted in few fixations of the contralesional line and a large ipsilesional bisection error, as seen in patients with real hemineglect [14], [15], [16]. Further, our top-down bisection task bias had to be weighted more strongly for hemineglect simulations to prevent the scanpath wandering away from the line. This suggests that parietal hemineglect patients may require greater top-down cognitive effort than healthy controls or hemianopic patients to maintain task-relevant eye movement and perform the task.

### 5. Line Length Estimation and Bisection

We simulated endstopping processes in V1 that detected the locations of line-ends during a line bisection task. Bisection performance in our lesion conditions mirrored that of healthy people if the decision about line length was determined by the endstopping process alone. However, integration of both V1 endstopping information and the perception of line length based on parietal activity was needed to accurately simulate pathologic line bisection errors. In hemianopia, propagation of activity in extrastriate cortex influences parietal cortex representation of line length and resulted in an over-estimation of the length of the contralesional line. In hemineglect there was an under-estimation of contralesional line length due to the parietal damage. Thus, our results suggest that the perceptual experience in patients supports integration of V1 endstopping input and activity in higher cortical areas, which occurs over the gathering of information during the evolution of the biased scanpath.

### 6. Predictions

Several interesting predictions follow from the proposal of these compensatory mechanisms. First, we modelled hemianopia resulting from a V1 lesion. However, it can also be caused by lesions elsewhere, notably the optic radiations and less frequently the optic tract or lateral geniculate nucleus. In cases where V1 is damaged, form completion processes must operate at a stage beyond V1 cortex, which we have modelled here in extrastriate cortex. However, if V1 is spared then lateral connectivity within V1 (such as that described by Grossberg & Raizada [24]) may contribute to form completion too. Therefore, we predict that hemianopic patients with an intact V1 will be more likely to experience the “hemianopic completion” phenomena where illusory percepts are based on lower level features being replicated in their blind fields, and possibly patients whose compensatory neural processes rely on higher cortex might experience more complex features being translated into the blind field.

Second, we demonstrated the effect of form completion into the blind field using only horizontally tuned neurons in extrastriate cortex and a simple horizontal stimulus. This same principle could be applied with lateral connections amongst neurons tuned to many different orientations. It will still be the case that neurons tuned to similar orientations will co-activate, whereas neurons tuned to orthogonal orientations will not, and may even inhibit each other. Hence, we predict that simple collinear forms with smooth or minimal orientation transitions can propagate into the blind field but complex shapes that contain edges of sharply changing orientations may not. This appears to be supported by the first empirical report of hemianopic completion [63], as mentioned above, but further investigation would be of interest.

Third, since activity due to lateral excitation takes time to propagate across extrastriate cortex and bias the parietal attention competition, saccades made after short fixation durations may be more likely to land on visible portions of the object in the intact hemifield, whereas saccades after longer fixation durations may have a better probability of landing on the unseen segment in the blind hemifield. Eye movement experiments in healthy controls have showed that slower “top-down” effects of target selection primarily influence saccades with longer latencies [77] and we have simulated these effects in our intact model [21]. It would be interesting to discover whether latency has a similar effect on the likelihood of fixating unseen versus seen portions of a stimulus in real hemianopic patients.

Fourth, during our simulated hemianopic line scanning, fixations in the blind field were accurately placed along the contralesional line and its virtual continuation. There was a slight vertical scatter above and below the line (figure 8b). We used a Gaussian kernel to convolve extrastriate activity from neighbouring cells (lateral connectivity), with the size of this kernel representing the retinotopic region over which lateral inputs are gained. Hence, the width determines the vertical “spreading” of activity within the extrastriate module. Given this, we predict that saccades by hemianopic patients to unseen segment of the line during bisection will have more vertical inaccuracy than saccade to visible segments.

Fifth, rehabilitative therapies that aim to restore visual fields typically involve visual stimulation (often luminance contrast dots) near the border of the scotoma, as for example with Vision Restoration Therapy [78]. It is possible that the repetitive stimulation of this area with visual form facilitates the strengthening of lateral connectivity in cortical areas responsive to this region. We predict that the use of oriented form in this region would be useful in stimulating continuation of form perception across the border of the scotoma.

### 7. Alternative Explanations

Our model has focused on determining if physiologically plausible cortical mechanisms can provide an account for specific line bisection and visual search behaviours in hemianopia and differences in the performance of these tasks between hemianopia and hemineglect. However, it is also possible that sub-cortical structures may have some contribution to the altered residual behaviour of these subjects. For example, in animal models of neglect, cortical lesions cause decreased ipsilateral and increased contralateral activity in the superior colliculus [79], though whether these tectal changes are independent determinants of behavior or merely reflections of altered cortical activity is not known. Also unknown is whether the changes in tectal activity in neglect differ from the effects of hemianopia alone. Subcortical contributions to vision also form the basis of some theories about blindsight, which postulate a tecto-pulvinar-cortical relay that can bypass striate cortex [80], [81] (not all blindsight may depend upon the superior colliculus, though: there may also be projections between the lateral geniculate nucleus and extrastriate cortex [82], [83]). While there are data showing that saccades to seen targets can be modified by blind-field stimuli [84], [85], evidence for saccadic localization of blind-field targets is mixed. Some studies fail to find saccadic localization without awareness [86], [87], [88], and in positive reports the correlation between saccadic amplitude and target position is usually weak and confined to paracentral locations [89], [90], [91], [92] or even reported for locations that recovered on subsequent perimetry [93]. More definitive evidence that tectal activity can support saccadic localization would come from blindsight studies in patients with hemispherectomies: however, few such saccadic studies have been done [94] and the validity of the general blindsight data in this population has been questioned in several reports [95], [96], [97], [98], [99], [100]. Finally, reports that have systematically examined larger series of patients instead of case studies have shown that blindsight visual abilities are rare, the exception rather than the rule in hemianopia [86], [101], [102]. Given this uncertainty about the role of the superior colliculus in blindsight, hemianopia and hemineglect, and in the interests of parsimony and simplicity, we chose to focus on modelling differences in cortical mechanisms as the most probable means of accounting for the contrast between hemianopia and hemineglect. However, we do not exclude the possibility that the superior colliculus could potentially contribute to saccadic guidance and supplement the processes we have described here, and could be included in more detailed future models of these conditions that aim at a more comprehensive account of perception and ocular motor behavior, beyond the focused question we ask regarding plausible cortical contributions to the relatively simple tasks of line bisection and visual search.

We have focused upon the role played by modified form grouping processes in hemianopic recovery and compensation for the blind field. We also demonstrated the effect of a simple top-down spatial bias to the blind field (gradually induced by rehabilitative training) and showed that this alone was insufficient to explain hemianopic behaviour. However, the role of other top-down or motor planning processes could have an important effect in directing recovery from lesion, particularly since training typically involves making strategic eye movements toward the blind field. In addition to the top-down spatial bias and stimulus-related grouping effects described here, some form of top-down systematic search strategy may additionally influence eye movements and search behaviour. Such a strategy could be context-dependent. Unlike that for hemineglect patients, hemianopic compensation likely benefits from an intact egocentric or retinotopic frame of reference when top-down search strategies are formed.

### Conclusions

We propose that at least two different methods of neural compensation might occur in hemianopia to facilitate scanning of the blind side. An increased spatial compensatory bias to the blind hemifield can account for the increased contralesional scanning of a large complex scene during visual search. However, this strategy alone fails to generate the contralesional increase in fixations during search and bisection and the small contralesional line bisection error seen in real hemianopia. Hence, we proposed a second compensatory mechanism, based on extrastriate lateral connectivity that provides perceptual grouping to enable Gestalt-like completion of form, a mechanism that can also explain the phenomenon of “hemianopic completion”. This mechanism propagates information from the intact to the blind hemifield so that the segment of an object in the blind hemifield can attract attention via subsequent parietal activation. This can explain why hemianopia patients make accurate fixations on previously unseen portions of a line during a bisection task, why they fixate beyond the end of the line, and why they show a small contralesional bisection error. Finally, we show that, while these compensatory mechanisms enable eye movements towards the blind side in hemianopia, they do not result in scanning of the contralesional side or correct the large ipsilesional bisection error in hemineglect. Hence the incorporation of these cortical compensatory features into our model provides an adequate account of the ocular motor and perception effects in two different tasks, visual search and line bisection, and how these are differentially affected by two different cerebral conditions, hemianopia from V1 lesions and hemineglect from parietal lesions.

## Supporting Information

Appendix S1 **Formal definition of the model.**
(PDF)
