# Supplementary material for: Visual Search and Line Bisection in Hemianopia: Computational Modelling of Cortical Compensatory Mechanisms and Comparison with Hemineglect
Source: PLoS One. 2013 Feb 4;8(2):e54919. doi: 10.1371/journal.pone.0054919 (PMC3563648; doi:10.1371/journal.pone.0054919)
Supplement: Appendix S1 — Formal definition of the model. (PDF) [file pone.0054919.s001.pdf]

Visual search and line bisection in hemianopia: computational modelling of cortical compensatory mechanisms and comparison with hemineglect

Linda J. Lanyon and Jason J. S. Barton

APPENDIX

This appendix provides details of the implementation of the model described in the main text. Note that equation numbering continues from the main text. The model is based on Lanyon and Denham [1,2]. Modules representing the extrastriate, inferior temporal and parietal areas interact dynamically to produce the attentional effects within the system. The retina and area V1 (primary visual cortex, striate cortex) do not form part of the dynamics of the system but provide featural input to the ventral stream via the extrastriate cortex. A shared Cartesian coordinate system is used to reference retinal position and receptive field position of cells in area V1, following the approach of Grossberg & Raizada (2000). Retinotopic cortical areas (parietal, extrastriate) use a different reference system that is also based on cartesian coordinates, similar to that used by Deco (2001). We assume transformation of spatial coordinates takes place, perhaps in parietal cortex, when relating retinal position to scene-based coordinates. We recognise the use of polar coordinate systems in some retinal models [3,4], but this is not currently a feature of our model.

A.1 Retina

Colour processing in the model focuses on the red-green channel with two colour arrays, Γ^{red} and Γ^{green}, a simplification of the output of the medium and long wavelength retinal cones, being input to the retinal ganglion cells. References to red and green throughout this paper refer to long and short wavelengths. The greyscale image, Γ^{grey}, used for form processing, is a composite of the colour arrays and provides luminance information.

A.1.1 Form processing in the Retina

At each location in the greyscale image, retinal ganglion broad-band cells perform simple centre-surround processing, according to Grossberg and Raizada (2000) as follows:

On-centre, off-surround broadband cells:

$$u^{+} = \Gamma_{ij}^{grey} - \sum_{pq} G_{pq}(i,j,\sigma_1)\Gamma_{pq}^{grey} \qquad [12]$$

Off-centre, on-surround broadband cells:

$$u^{-} = -\Gamma_{ij}^{grey} + \sum_{pq} G_{pq}(i,j,\sigma_1)\Gamma_{pq}^{grey} \qquad [13]$$

where $G_{pq}(i,j,\sigma_1)$ is a 2-dimensional Gaussian kernel, given by:

$$G_{pq}(i,j,\sigma_1) = \frac{1}{2\pi\sigma_1^2}\exp\left(-\frac{1}{2\sigma_1^2}\left((p-i)^2 + (q-j)^2\right)\right) \qquad [14]$$

The Gaussian width parameter is set to: $\sigma_1 = 1$

These broadband cells provide luminance inputs to V1 interblob simple cells that are orientation selective.

A.1.2 Colour processing in the Retina

Retinal concentric single opponent cells process colour information as follows:

Red on-centre, off-surround concentric single-opponent cells:

$$v_{ij}^{redON} = \Gamma_{ij}^{red} - \sum_{pq} G_{pg}(i,j,\sigma_1)\Gamma_{pq}^{green} \qquad [15]$$

Red off-centre, on-surround concentric single-opponent cells:

$$v_{ij}^{redOFF} = -\Gamma_{ij}^{red} + \sum_{pq} G_{pg}(i,j,\sigma_1)\Gamma_{pq}^{green} \qquad [16]$$

Green on-centre, off-surround concentric single-opponent cells:

$$v_{ij}^{greenON} = \Gamma_{ij}^{green} - \sum_{pq} G_{pg}(i, j, \sigma_1)\Gamma_{pq}^{red} \quad [17]$$

Green off-centre, on-surround concentric single-opponent cells:

$$v_{ij}^{greenOFF} = -\Gamma_{ij}^{green} + \sum_{pq} G_{pg}(i, j, \sigma_1)\Gamma_{pq}^{red} \quad [18]$$

These concentric single-opponent cells provide colour-specific inputs to V1 double-opponent blob neurons

A.2 Area V1

The V1 module consists of K + C neurons at each location in the original image, so that neurons detect K orientations and C colours. The size of filters used in V1 determines the ratio of pixels to degrees of visual angle so that V1 receptive fields cover approximately 1° of visual angle [5].

A.2.1 Form Processing in V1

For orientation detection, V1 simple and complex cells are modelled as described by Grossberg & Raizada [6], with the distinction that two spatial resolutions are calculated here. Simple cells detect oriented edges using a difference-of-offset-Gaussian (DOOG) kernel where the right and left-hand kernels of the simple cells are given by:

$$R_{rijk} = \Sigma_{pq}([u^{+}_{pq}]_{+} - [u^{-}_{pq}]_{+})\ [D^{(rk)}_{pqij}]_{+} \quad [19]$$

$$L_{rijk} = \Sigma_{pq}([u^{-}_{pq}]_{+} - [u^{+}_{pq}]_{+})\ [-D^{(rk)}_{pqij}]_{+} \quad [20]$$

where:

u^{+} and u^{-} are the outputs of the retinal broadband cells above

$[x]_{+}$ signifies half-wave rectification, i.e. $[x]_{+} = x$ if $x \geq 0$; 0 otherwise

The oriented DOOG filter $D^{(lk)}_{pqij}$ is given by:

$D^{(rk)}_{pqij} = G_{pq}(i - \delta\cos\theta, j - \delta\sin\theta, \sigma_2) - G_{pq}(i + \delta\cos\theta, j + \delta\sin\theta, \sigma_2)$ [21]

where:

$\delta = \sigma_2/2$ and $\theta = \pi(k-1)/K$, where k ranges from 1 to 2K, K being the total number of orientations (2 is used here).

σ_2 is the width parameter for the DOOG filter, set as below

r is the spatial frequency octave (i.e. spatial resolution), such that

r = 1 and σ_2 = 1.2 for high resolution processing (in the parvocellular pathway; The model also comprises a magnocellular pathway, where r = 2 and σ_2 = 2.2 for low resolution processing, not discussed here)

$G_{pq}(i, j, \sigma_1)$ is a 2-dimensional Gaussian kernel, given by:

$$G_{pq}(i, j, \sigma_1) = \frac{1}{2\pi\sigma_1^2}\exp\left(-\frac{1}{2\sigma_1^2}\left((p-i)^2 + (q-j)^2\right)\right) \quad [22]$$

The direction-of-contrast sensitive simple cell response is given by:

$S_{rijk} = \gamma[R_{rijk} + L_{rijk} - |R_{rijk} - L_{rijk}|]_+$ [23]

γ is set to10

The complex cell response is invariant to direction of contrast and is given by:

$I_{rijk} = S_{rijk} + S_{rij(k+K)}$ where k ranges from 1 to K [24]

The value of the complex cells, I_{rijk}, over the area of the current retinal image, is input to the extrastriate module.

A.2.2 Colour Processing in V1

The outputs of LGN concentric single-opponent cells (simplified to be the retinal cells here) are combined in the cortex in the double-opponent cells concentrated in the blob zones of layers 2 and 3 of V1, which form part of the parvocellular system. The outputs of blob cells are transmitted to the thin stripes of V2 and from there to colour-specific neurons in the extrastriate module. For simplicity, V2 is not included in this model.

Double-opponent cells have a centre-surround antagonism and combine inputs from different single-opponent cells as follows:

Red on-centre portion: [25]

$$\omega_{ij}^{redON} = \sum_{pq} G_{pq}(i,j,\sigma_1)v_{ij}^{redON} + \sum_{pq} G_{pq}(i,j,\sigma_1)v_{ij}^{greenOFF} - \sum_{pq} G_{pq}(i,j,\sigma_1)v_{ij}^{redOFF} - \sum_{pq} G_{pq}(i,j,\sigma_1)v_{ij}^{greenON}$$

Red off-surround portion: [26]

$$\omega_{ij}^{redOFF} = \sum_{pq} G_{pq}(i,j,\sigma_2)v_{ij}^{redOFF} + \sum_{pq} G_{pq}(i,j,\sigma_2)v_{ij}^{greenON} - \sum_{pq} G_{pq}(i,j,\sigma_2)v_{ij}^{redON} - \sum_{pq} G_{pq}(i,j,\sigma_2)v_{ij}^{greenOFF}$$

Green on-centre portion: [27]

$$\omega_{ij}^{greenON} = \sum_{pq} G_{pq}(i,j,\sigma_1)v_{ij}^{greenON} + \sum_{pq} G_{pq}(i,j,\sigma_1)v_{ij}^{redOFF} - \sum_{pq} G_{pq}(i,j,\sigma_1)v_{ij}^{greenOFF} - \sum_{pq} G_{pq}(i,j,\sigma_1)v_{ij}^{redON}$$

Green off-surround portion: [28]

$$\omega_{ij}^{greenOFF} = \sum_{pq} G_{pq}(i,j,\sigma_2)v_{ij}^{greenOFF} + \sum_{pq} G_{pq}(i,j,\sigma_2)v_{ij}^{redON} - \sum_{pq} G_{pq}(i,j,\sigma_2)v_{ij}^{greenON} - \sum_{pq} G_{pq}(i,j,\sigma_2)v_{ij}^{redOFF}$$

where:

σ_1=1.2, σ_2=1.5

The complete red-selective blob cell is given by:

$$I_{ijc^1} = \gamma\left[\omega_{ij}^{redON} - \omega_{ij}^{redOFF}\right]_+ \quad [29]$$

The complete green-selective blob cell is given by:

$$I_{ijc^2} = \gamma\left[\omega_{ij}^{greenON} - \omega_{ij}^{greenOFF}\right]_+ \quad [30]$$

where:

$\gamma = 0.2$ This scales the output of V1 blob cells to be consistent with that of the orientation-selective cells

$c^1 = K + 1$ This represents the position of the first colour input to the extrastriate module (i.e. red)

$c^2 = K + 2$ This represents the position of the second colour input to the extrastriate module (i.e. green)

The blob cell outputs over the area of the current retinal image are input to the extrastriate module.

A.3 Dynamic Cortical Modules

The dynamic cortical modules (extrastriate, parietal and IT) follow a similar approach to that described by Deco [7,8] and Usher and Niebur [9] and use mean field population dynamics [10,11]. The response function, which transforms current (activity within the assembly) into discharge rate, is given by the following sigmoid function that has a logarithmic singularity [10]:

$$F(x) = \frac{1}{T_r - \tau \log(1 - \frac{1}{\tau x})} \qquad [31]$$

where:

T_r, the absolute refractory time, is set to 1ms

τ, is the membrane time constant (where $\frac{1}{\tau}$ determines the cell's firing threshold). The threshold is normally set to half the present maximum activity in the layer (in the extrastriate module, this is half the maximum *within the feature type* in order that the object-based attention differences between features are not lost due to the normalising effect of this function)

The parietal module contains the same number of neurons as the extrastriate module and has reciprocal connections with both the orientation and colour layers in the extrastriate module. The size of the extrastriate and parietal modules is determined by the size of the retinal image, which is flexible. For monochromatic simulations the extrastriate module contains only orientation selective assemblies.

The dynamic portion of the model is run such that the differential equations are solved numerically by running simulations in computer software. The differential equations are implemented as difference equations so that the activity of neurons is updated in the software program at each time step. The order of updates is parietal, extrastriate, IT. Outputs from the system are robust across reasonable step sizes: we typically use 1ms for simulating single cell responses and 5ms for larger scanpath simulations such as those demonstrated here.

A.3.1 Extrastriate Module

The extrastriate module consists of a three dimensional matrix of pyramidal cell assemblies. The first two dimensions represent the retinotopic arrangement and the other represents the feature types. In the latter dimension, there are K + C layers of cell assemblies, as shown in figure S1b: K layers are each selective for an orientation (normally vertical and horizontal), C layers are each selective for a particular colour (normally red and green). Two sets of inhibitory interneuron pools mediate competition between orientations and between colours respectively. The extrastriate module receives convergent input from V1 over the area of its receptive field with a latency of 60ms to reflect normal response latencies [12].

A.3.1.1 Form processing in the extrastriate module

The output from the V1 simple cell process, I_{ijk}, for each position (i,j) at orientation k, provides the bottom-up input to orientation selective pyramidal assemblies in the extrastriate module that evolve according to the following dynamics:

$$\tau_1 \frac{\delta}{\delta t} W_{ijk}(t) = -W_{ijk}(t) + \alpha F\left(W_{ijk}(t)\right) - \beta F\left(W_{ij}^{IK}(t)\right) + \chi \sum_{pq} I_{pqk}(t) + \\ + \gamma F\left(Y_{ij}(t)\right) + \eta \sum_{m} B_{W_{ijk} X_m} . F(X_m(t)) + I_0 + \nu \qquad [32]$$

where:

τ_1 is set to 20ms

α is the weight of excitatory input from other cells in the pool, set to 0.95

β is the weight of inhibitory interneurons input, set to 10

I_{pqk} is the input from the V1 simple cell edge detection process at all positions within the extrastriate module receptive field area (p,q; which differs from the p,q used to denote the area of the Gaussian above), and of preferred orientation k

The receptive field area (p,q) used in the extrastriate and parietal modules, is mapped into V1 coordinates (x,y) as follows:

Starting position of V1 inputs to extrastriate cortex (rows), V1startRow =

((i-1) * (exRFsize- exRFoverlap))+1 [33]

Ending position of V1 inputs to extrastriate cortex (rows) = V1startRow + exRFsize -1

Starting position of V1 inputs to extrastriate cortex (columns), V1startCol =

((i-1) * (exRFsize- exRFoverlap))+1 [34]

Ending position of V1 inputs to extrastriate cortex (columns) = V1startCol + exRFsize -1

where:

exRFsize, the size of the extrastriate receptive field in terms of V1 inputs = 23

exRFoverlap, the amount of overlap in extrastriate receptive fields in terms of V1 inputs, = 1

χ is the weight of V1 inputs, set to 5

Y_{ij} is the input from the parietal module, reciprocally connected to the extrastriate module

γ is the weight of parietal inputs, set to 3

X_m is the feedback from IT cell populations via weight $B_{W_{ijk} X_m}$, described later

η is the parameter representing the strength of object-related feedback from IT; set to 5

I_0 is a background current injected in the pool, set to 0.25

ν is additive noise, which is randomly selected from a uniform distribution on the interval (0,0.1)

The dynamic behaviour of the associated inhibitory pool for orientation-selective cell assemblies in the extrastriate module is given by:

$$\tau_1 \frac{\delta}{\delta t} W_{ij}^{IK}(t) = -W_{ijk}^{IK}(t) + \lambda \sum_k F\left(W_{ijk}(t)\right) - \mu F\left(W_{ij}^{IK}(t)\right) \quad [35]$$

where:

τ_1 is set to 20ms

λ is the weight of pyramidal cell assembly input, set to 1

μ is the weight of inhibitory interneuron input, set to 1

Over time, this results in local competition between different orientation selective cell assemblies.

A.3.1.2. Colour processing in the extrastriate module

The output from the V1 simple cell process, I_{ijc}, for each position (i,j) and colour c, provides the bottom-up input to colour selective pyramidal assemblies in the extrastriate module that evolve according to the following dynamics:

$$\tau_1 \frac{\delta}{\delta t} W_{ijc}(t) = -W_{ijc} + \alpha F\left(W_{ijc}(t)\right) - \beta F\left(W_{ij}^{IC}(t)\right) + \chi \sum_{pq} I_{pqc}(t) + \gamma F\left(Y_{ij}(t)\right) + \eta \sum_m B_{W_{Iijc} X_m} . F(X_m(t)) + I_0 + \nu \quad [36]$$

where:

I_{pqc} is the input from the V1 blob cells at all positions within the extrastriate module receptive field area (p,q), and of preferred colour c

X_m is the feedback from IT cell populations via weight $B_{W_{ijc} X_m}$, described later

The remaining terms are the same as those in equation 32.

The dynamic behaviour of the associated inhibitory pool for colour-selective cell assemblies in the extrastriate module is given by:

$$\tau_1 \frac{\delta}{\delta t} W_{ij}^{IC}(t) = -W_{ijc}^{IC}(t) + \lambda \sum_c F\left(W_{ijc}(t)\right) - \mu F\left(W_{ij}^{IC}(t)\right) \qquad [37]$$

Parameters take the same values as those in equation 35.

Over time, this results in local competition between different colour selective cell assemblies.

A.3.2 Parietal Module

In the intact model, the pyramidal cell assemblies in the parietal module evolve according to the following dynamics:

$$\tau_1 \frac{\delta}{\delta t} Y_{ij}(t) = -Y_{ij}(t) + \alpha F\left(Y_{ij}(t)\right) - \beta F\left(Y^I(t)\right) + \chi \sum_k F\left(W_{ijk}(t)\right) + \varepsilon \sum_c F\left(W_{ijc}(t)\right)$$
$$+ \gamma P_{ij}^d(t) + \eta \sum_{pq} Z_{pq} + I_0 + \nu \qquad [38]$$

where:

τ_1 is set to 20ms

α is the weight of excitatory input from other cells in the pool, set to 0.95

β is the weight of inhibitory input, set to 1

W_{ijk} is the orientation input from the extrastriate module for orientation k, at location (i,j). Extrastriate inputs are normalised by dividing the total input to each parietal assembly by the total number of active (i.e. non-zero) inputs, as follows:

$$\sum_k F\left(W_{ijk}(t)\right) = \sum_k F\left(W_{ijk}(t)\right) / \sum_k \left[F\left(W_{ijk}(t)\right)\right]_+ \qquad [39]$$

where $[x]_+$ signifies a step function rectification, such that $[x]_+ = 1$ if $x \geq 0$; 0 otherwise

χ the weight of orientation input, is set to 3.5

W_{ijc} is the colour input from the extrastriate module for colour c, at location (i,j)

Extrastriate inputs are normalised by dividing the total input to each parietal assembly by the total number of active (i.e. non-zero) inputs, as follows:

$$\sum_k F\left(W_{ijk}(t)\right) = \sum_k F\left(W_{ijk}(t)\right) \;/\; \sum_k \left[F\left(W_{ijk}(t)\right)\right]_+ \qquad [40]$$

where $[x]_+$ signifies a step function rectification, such that $[x]_+ = 1$ if $x \geq 0$; 0 otherwise

ε the weight of colour input, is set to 2

P_{ij}^d is the spatial Attention Window (AW) bias injected directly into this pool when there is a requirement to attend to this spatial location following fixation. This bias is set to 1 within the AW and zero outside.

γ is the weight of the spatial AW bias, set to 2.5

Z_{pq} is the bias from area pq of the novelty map (which is the size of the original image, N). Area pq represents the size of the parietal receptive field, as specified for the extrastriate module above. (see Lanyon & Denham [2] for information about the novelty bias)

η is the weight of the novelty bias, normally set to 0.0009

I_0 is a background current injected in the pool, set to 0.25

ν is additive noise, which is randomly selected from a uniform distribution on the interval (0,0.1)

The dynamic behaviour of the associated inhibitory pool in the parietal module is given by:

$$\tau_1 \frac{\delta}{\delta t} Y^I(t) = -Y^I(t) + \lambda \sum_{ij} F\left(Y_{ij}(t)\right) - \mu F\left(Y^I(t)\right) \qquad [41]$$

where:

τ_1 is set to 20ms

λ is the weight of pyramidal cell assembly input, set to 1

μ is the weight of inhibitory interneuron input, set to 1

Over time, this results in competition between neurons in the parietal module.

A.3.3 IT

The model IT encodes all possible objects and receives feedforward feature inputs from the extrastriate module with a latency of 80ms to reflect normal response latencies [13]. IT also feeds back an object bias to extrastriate cells. The strength of these connections is given by the following weights, which are set by hand (to –1 or 0, as appropriate, for inhibitory feedback, although the model may also be implemented with excitatory feedback; 0, +1) to represent prior object learning. These simple matrices reflect the type of weights that would be achieved through Hebbian learning without the need for a lengthy learning procedure (such as [7]), which is not the aim of this work. The result is that the connections that are active for excitatory feedback (or inactive for inhibitory feedback) are those features relating to the object.

Extrastriate Cell Assemblies to IT (Feedforward)

$A_{X_m W_{ijz}}$

IT to Extrastriate Cell Assemblies (Feedback)

$B_{W_{ijz} X_m}$ = -1 for features that are not associated with this object, 0 otherwise

where z indicates orientation, k, or colour, c

The pyramidal cell assemblies in IT evolve according to the following dynamics:

$$\tau_1 \frac{\delta}{\delta t} X_m(t) = -X_m(t) + \alpha F(X_m(t)) - \beta F\left(X^I(t)\right) + \chi \sum_{ijk} A_{X_m W_{ijk}} . F\left(W_{ijk}(t)\right) + \chi \sum_{ijc} A_{X_m W_{ijc}} . F\left(W_{ijc}(t)\right) + \gamma P_M^v(t) + \gamma P_M^{vBackground}(t) + I_0 + \nu \qquad [42]$$

where:

τ_1 is set to 20ms

β is the weight of inhibitory interneuron input, set to 0.01

W_{ijk} is the feedforward input from the extrastriate module relating to orientation information, via weight $A_{X_m W_{ijk}}$

Extrastriate inputs are normalised by dividing the total input to each parietal assembly by the total number of active (i.e. non-zero) inputs, as follows:

$$\sum_{ijk} A_{X_m W_{ijk}} .F\left(W_{ijk}(t)\right) = \sum_{ijk} A_{X_m W_{ijk}} .F\left(W_{ijk}(t)\right) \;/\; \sum_{ijk} \left\lfloor A_{X_m W_{ijk}} .F\left(W_{ijk}(t)\right)\right\rfloor_{+} \qquad [43]$$

where $[x]_+$ signifies a step function rectification, such that $[x]_+ = 1$ if $x \geq 0$; 0 otherwise

W_{ijc} is the feedforward input from the extrastriate module relating to colour information, via weight $A_{X_m W_{ijc}}$

Extrastriate inputs are normalised by dividing the total input to each parietal assembly by the total number of active (i.e. non-zero) inputs, as follows:

$$\sum_{ijc} A_{X_m W_{ijc}} .F\left(W_{ijc}(t)\right) = \sum_{ijc} A_{X_m W_{ijc}} .F\left(W_{ijc}(t)\right) \;/\; \sum_{ijc} \left\lfloor A_{X_m W_{ijc}} .F\left(W_{ijc}(t)\right)\right\rfloor_{+} \qquad [44]$$

where $[x]_+$ signifies a step function rectification, such that $[x]_+ = 1$ if $x \geq 0$; 0 otherwise

χ is the weight of extrastriate inputs, set to 2.5

γ is the weight of the object-related bias from prefrontal cortex, set to 1.2

P_M^v is the object-related feedback current from ventrolateral prefrontal cortex, injected directly into this pool

This feedback is sigmoidal over time as follows:

For the target object:

$P_M^v = 0$

Other objects receive inhibitory feedback as follows:

$P_M^v = -1/(1+\exp(\tau_{sig}-t))$ [45]

where t = time (in milliseconds) and

τ_{sig} is the point in time where the sigmoid reaches half its peak value: Set to 150ms in order to accurately replicate the time course of attentional effects in individual cells [1].

The remaining terms and parameters are evident from previous equations.

The dynamic behaviour of the associated inhibitory pool in IT is given by:

$$\tau_1 \frac{\delta}{\delta t} X^I(t) = -X^I(t) + \lambda \sum_m F\left(X_m(t)\right) - \mu F\left(X^I(t)\right) \quad [46]$$

where:

τ_1 is set to 20ms

λ is the weight of pyramidal cell assembly input, set to 3

μ is the weight of inhibitory interneuron input, set to 1

References

1. Lanyon LJ, Denham SL (2009) Modelling attention in individual cells leads to a system with realistic saccade behaviours. Cogn Neurodyn 3: 223-242.
2. Lanyon LJ, Denham SL (2004) A model of active visual search with object-based attention guiding scan paths. Neural Netw 17: 873-897.
3. Balasubramanian M, Polimeni J, Schwartz EL (2002) The V1 -V2-V3 complex: quasiconformal dipole maps in primate striate and extra-striate cortex. Neural Netw 15: 1157-1163.
4. Fazl A, Grossberg S, Mingolla E (2009) View-invariant object category learning, recognition, and search: how spatial and object attention are coordinated using surface-based attentional shrouds. Cogn Psychol 58: 1-48.
5. Wallis G, Rolls ET (1997) Invariant face and object recognition in the visual system. Prog Neurobiol 51: 167-194.
6. Grossberg S, Raizada RD (2000) Contrast-sensitive perceptual grouping and object-based attention in the laminar circuits of primary visual cortex. Vision Res 40: 1413-1432.
7. Deco G (2001) Biased competition mechanisms for visual attention in a multimodular neurodynamical system. . Emergent Neural Computational Architectures Based on Neuroscience: Towards Neuroscience-Inspired Computing 2036: 114-126.
8. Deco G, Lee TS (2002) A unified model of spatial and object attention based on inter-cortical biased competition. Neurocomputing 44: 775-781.
9. Usher M, Niebur E (1996) Modeling the temporal dynamics of IT neurons in visual search: A mechanism for top-down selective attention. Journal of Cognitive Neuroscience 8: 311-327.
10. Gerstner W (2000) Population dynamics of spiking neurons: fast transients, asynchronous states, and locking. Neural Comput 12: 43-89.
11. Wilson HR, Cowan JD (1972) Excitatory and Inhibitory Interactions in Localized Populations of Model Neurons. Biophysical Journal 12: 1-&.
12. Luck SJ, Chelazzi L, Hillyard SA, Desimone R (1997) Neural mechanisms of spatial selective attention in areas V1, V2, and V4 of macaque visual cortex. Journal of Neurophysiology 77: 24-42.
13. Wallis G, Rolls ET (1997) Invariant face and object recognition in the visual system. Progress in Neurobiology 51: 167-194.
